# Antibiotic Azithromycin inhibits brown/beige fat functionality and promotes obesity in human and rodents

**DOI:** 10.7150/thno.63067

**Published:** 2022-01-01

**Authors:** Jian Yu, Xin Chen, Yuanjin Zhang, Xiangdi Cui, Zhe Zhang, Wenxiu Guo, Dongmei Wang, Shengbo Huang, Yanru Chen, Yepeng Hu, Cheng Zhao, Jin Qiu, Yu Li, Meiyao Meng, Mingwei Guo, Fei Shen, Mengdi Zhang, Ben Zhou, Xuejiang Gu, Jiqiu Wang, Xin Wang, Xinran Ma, Lingyan Xu

**Affiliations:** 1 Shanghai Key Laboratory of Regulatory Biology, Institute of Biomedical Sciences and School of Life Sciences, East China Normal University, Shanghai, 200241, China.; 2 Department of Endocrinology and Metabolism, China National Research Center for Metabolic Diseases, Ruijin Hospital, Shanghai Jiao Tong University School of Medicine, Shanghai, 200025, China; 3 Department of Endocrine and Metabolic Diseases, the First Affiliated Hospital of Wenzhou Medical University, Wenzhou, Zhejiang, 325000, China.; 4 Key Laboratory of Adolescent Health Assessment and Exercise Intervention, Ministry of Education, East China Normal University, Shanghai, China.; 5 Key Laboratory of Nutrition and Metabolism, Institute for Nutritional Sciences, Shanghai Institutes for Biological Sciences, Chinese Academy of Sciences, Shanghai, 200031, China.

**Keywords:** Obesity, Azithromycin, Adipose tissue, Energy metabolism, Oxidative damage

## Abstract

Obesity, a metabolic disease caused by multiple factors, has become a global health problem. In addition to nutrient intake and sedentary lifestyle, environmental pollutants exposure has been shown to be involved in obesity epidemics. Antibiotics, a new type of environmental pollutant, have been widely used in animal husbandry, aquaculture and microorganism. However, the effects of antibiotics exposure on fat metabolism and metabolic diseases are largely unknown.

**Methods:** We screened major types of antibiotics to examine their effects on the differentiation capacity and thermogenic functionality of brown and beige adipocytes, and found that azithromycin, one major kind of macrolide antibiotics suppressed brown and beige adipocyte functionality. We thus examined azithromycin accretion in adipose tissues of obese patients that correlates with BMI by high performance liquid chromatography-tandem mass spectrometry and systematically explore the influences of azithromycin on adiposity and metabolic performance in mice under high diet.

**Results:** Azithromycin (macrolides) inhibits the mitochondrial and thermogenic gene programs of brown and beige adipocytes, thus disrupting their mitochondrial function and thermogenic response. Consistently, azithromycin treatment are more prone to diet-induced obesity in mice, and this was associated with impaired energy expenditure. Importantly, azithromycin is more accumulated in adipose tissue of obese patients and correlates with BMI and body weight. Mechanistically, we found that azithromycin inhibits mitochondria respiratory complex I protein levels and increases reactive oxidative species (ROS) levels, which causes damage of mitochondrial function in brown and beige adipocytes. The deleterious effects of azithromycin can be ameliorated by antioxidant N-acetyl-L-cysteine.

**Conclusions:** Taken together, this work highlights the possible role of azithromycin in obesity epidemic and presents strategies for safe applications of antibiotics in the future.

## Introduction

The global obesity epidemic poses great health challenges worldwide for its close link to metabolic disorders, including type 2 diabetes, fatty liver, cardiovascular diseases and certain types of cancer 1, 2. The excessive nutrient intake, sedentary lifestyle and deteriorating environments of the modern society contributed to the rising prevalence of obesity 3. For example, with the advancement of industry development comes the wide use of industrial products in daily life, which brings not only convenience, but also environmental pollutants originated from the chemicals used in the manufacture of these products. It has been demonstrated that environmental pollutants are tightly involved in the onset of obesity and metabolic diseases 4. One of the examples is endocrine disruptor bisphenol A (BPA) and polybrominated diphenyl. As major ingredients in dry cleaning reagent, pesticide and plastics, they have been shown to pose significant threats to metabolic health in humans 5-8. Besides, we recently reported that silver nanoparticles, commonly used in water refreshment systems, daily necessities and biosensors, promote adiposity by inhibiting beige adipocyte differentiation and functionality in mice 9. As a part of our everyday life, large populations are potentially exposed to the threat of environmental pollutants, emphasizing the importance of studying impacts and underlying mechanisms of environmental pollutants to the pathogenesis of metabolic diseases.

Since the discovery of penicillin in 1928, the large family of antibiotics have saved countless lives from severe infections and greatly promoted human health 10. Nowadays, as a low-cost, highly effective means against a broad spectrum of bacterial infections, antibiotics are regularly used, even abused, in clinic as well as in animal husbandry and aquaculture 11. However, it has been gradually recognized that the public health is threatened not only by clinical antibiotic abuse 12, but also by the wide antibiotic abuse in livestock and aquaculture businesses, which caused significant antibiotics residues in meat and aquatic products, as well as in land and waters 13, 14. These residual antibiotics find their ways into the human body through daily food/water consumption or environment exposure, thus have become a new type of environmental pollutants that possess threats to human health, with special relevant to chronic metabolic diseases 15. For example, antibiotic abuse disrupts gut microbiome and in turn leads to obesity, which underlies the similar geographical distribution between high obesity incidence and wide macrolides prescriptions in US 16, 17. This was recapitulated in mouse models since data showed that subtherapeutic or low doses of antibiotics lead to altered gut microbiota community and weight gain in mice 18. Moreover, it is reported that penicillin treatment on mice in pregnancy increased the risk of obesity in offspring through lactation, suggesting that residual antibiotics also impact metabolic health by means of ingestion 19, 20.

Obesity is an excessive accumulation of fat, and the metabolic dysfunctions in adipocytes are central to obesity development 21. Different types of adipocytes have unique contributions toward metabolic health, i.e. white adipocytes are major site for energy storage, while brown and beige adipocytes are major contributors to energy expenditure 22, 23. Brown and beige adipocytes exist in human adults and their functionality were decreased in obese patients 24, 25. As thermogenic-poised adipocytes, brown and beige adipocytes were enriched with mitochondrial in their basal conditions, while cold or β-adrenergic stimulation multiplies their mitochondrial contents for heat production 26. Notably, many antibiotics function by binding to bacterial ribosomes and disrupt its normal functions, i.e. nascent peptide exit, tRNA binding, peptidyl transferase targeting, etc. This results in mismatch of amino acids, hindered translocation and premature dissociation of peptidyl tRNA from ribosomes, which reduced ribosomal protein translation, and eventually impact ribosome assembly and cause lethal defects in bacterial protein synthesis 27, 28. It has been well accepted that mitochondrial evolutionally have a bacterial ancestry. Though much smaller than that of bacteria, mitochondrial ribosomes share high structural similarity in critical functional regions with bacterial ribosomes, thus allows potential off-target effects of some antibiotics on mitochondrial and result in inhibition of mitochondrial translation and normal functionality 29. This is of special relevance to brown and beige adipocytes that rely on high mitochondrial quantity and quality for physiological functions, which predisposes them to vulnerability to antibiotics interference. Indeed, studies have shown that bactericidal antibiotics may lead to mitochondrial dysfunction in mammalian cells 30. Besides, it has been reported that antibiotics including norfloxacin, ampicillin, enoxacin, doxycycline and streptomycin are closely related to obesity progression via their influences on microbiota, adipose tissue miRNA, mitochondrial respiration, inflammatory cytokines and MMPs, TRP channels and calcium-mitochondrial axis, etc., suggesting the broad and diverse influences of antibiotics on fat biology and obesity 31-36. However, the exact metabolic impacts of specific antibiotics on thermogenic adipocyte functions and the pathogenesis of obesity are not well understood.

Considering the correlation of antibiotic abuse and obesity epidemics, we screened and assessed the influences of antibiotic exposure on brown and beige adipocyte functionality, as well as metabolic performances in mice under high fat diet. Our results suggested that azithromycin, one major kind of macrolide antibiotics with wide applications in clinic and in livestock/aquaculture raising etc., suppressed brown and beige adipocyte functionality via inhibition of mitochondrial complex components, which generates elevated ROS signaling and results in increased obesity and metabolic dysfunctions in mice. The adverse metabolic effects of azithromycin could be ameliorated by anti-oxidant NAC treatment. Importantly, we found increased accumulation of azithromycin in adipose tissues of obese patients compared to lean subjects, suggesting that azithromycin usage might pose direct interference on brown and beige adipocytes functionality, thus contributing to the obesity epidemics.

## Materials and methods

### Cell culture

Immortal brown and beige preadipocytes were generously provided by Professor Dongning Pan (Fudan University Shanghai Medical College) and Qiurong Ding (Chinese Academy of Sciences) and cultured with Dulbecco's Modified Eagle Medium (DMEM) supplemented 20% fetal bovine serum (FBS) and 1% penicillin/streptomycin (P/S) in a humidified incubator at 37 ℃ with 5% CO_2_. When preadipocytes reached confluent, cells were differentiated under induction medium for 48 h (DMEM with 10% FBS, 1% P/S, insulin 1 μg/mL (Eli Lilly, USA), 3-isobutyl-1-methylxanthine (IBMX, Sigma-Aldrich) 0.5 mmol/L, T3 1 nmol/L (Sigma-Aldrich), dexamethasone 1 μmol/L (Sigma-Aldrich) and rosiglitazone 1 μmol/L) (Sigma-Aldrich) and changed to maintenance medium every two days (Brown adipocytes: DMEM with 10% FBS, 1% P/S, insulin 1 μg/mL, IBMX 0.5 mmol/L and T3 1 nmol/L. Beige adipocytes: DMEM with 10% FBS, 1% P/S, insulin 5 μg/mL and rosiglitazone 1 μmol/L).

Primary adipocytes were isolated from mice subcutaneous fat and brown fat and digested with collagenase at 37 ℃ for 20-30 min 37. After digestion termination with DMEM containing 10% FBS, the released cells were filtered through 200-μm polypropylene filters and centrifuged at the speed of 3000 rpm for 5 min then resuspended and planted in wells. The isolated SVFs from BAT and iWAT were differentiated and cultured with medium containing 10% FBS, 1% P/S supplemented with insulin 5 μg/mL, indomethacin 0.5 mmol/L, dexamethasone 1 μmol/L, T3 1 nmol/L, and rosiglitazone 1 μmol/L for 48 h and subsequently cultured in maintenance medium (insulin 5 μg/mL and T3 1 nmol/L) until day 8.

### Antibiotics treatment

Antibiotics (Azithromycin, Doxycycline, Oxytetracycline, Cefalexin, Penicillin G, Sulfadiazine, Sulfathiazole, Norfloxacin, Oxolinic Acid, Chloramphenicol, G418, Erythromycin, Roxithromycin and Telithromycin purchased from MedChemExpress MCE compound library were dissolved by dimethyl-sulfoxide (DMSO) and treated beige or brown adipocytes at 5 μM. These antibiotics and their controls (10% DMSO in saline) were also unilaterally injected into iWAT of mice at 1 mg/kg.

Azithromycin for *in vivo* experiments was purchased from Sigma-Aldrich (United States Pharmacopeia (USP) Reference Standard). For delivery of antibiotics and antioxidant via drinking water, AZI (50 mg/kg/day) and Antioxidant N-Acetyl-L-cysteine (NAC, 1.5 g/kg/day) were used at previously reported dosage without adjusting pH 30, 38. In order to eliminate the influence of gut microbiota on host metabolism, mice were given drinking water with mixed antibiotics containing ampicillin (Sigma-Aldrich) and neomycin (MCE, China) both at 1.0 g/L concentration (ABX) 7 days before AZI treatment to remove intestinal flora. ABX was present in drinking water along with AZI or control throughout the experimental process 39. In summary, HFD mice were given ABX (1.0 g/L), ABX+AZI (50 mg/kg/day) or ABX+AZI+NAC (1.5 g/kg/day) via drinking water, which were replaced every three days. Besides, mice were also treated with control or AZI (50 mg/kg) via intravenous (i.v.) injection acutely three times on Monday, Wednesday and Friday, as well as with control, AZI (50 mg/kg) or AZI (50 mg/kg)+NAC (100 mg/kg) intraperitoneal (i.p.) injection chronically on Monday, Wednesday and Friday every week for 10 weeks. NAC and ZLN005 (MCE) were treated in adipocytes at 5 mM and 10 μM as previously reported 35, 40.

### Lipid staining and Oil Red O quantitative analyses

For oil red O staining, adipocytes were washed twice with phosphate buffer saline (PBS) and fixed with 10% neutral formalin for 20 min at room temperature (RT). Subsequently, the cells were rinsed with 60% isopropyl alcohol and stained with oil red O solution (Sigma-Aldrich) for 20 min. After taking picture, Oil red O was eluted with 60% isopropanol and measured at 520 nm by SpectraMax 190 microplate reader (Molecular Devices).

### Cell viability assay

Cell viability was assessed by the cell counting kit-8 (CCK-8) (Beyotime, China). Briefly, adipocytes were incubated for 2 h with CCK-8 solution at 37 ℃, and the absorbance was measured at 450 nm on a SpectraMax 190 microplate reader (Molecular Devices).

### Real-time PCR and transcriptome sequencing analysis

Total RNA was isolated from frozen tissues (human subjects and mice) or cultured cells with RNAiso Plus (Takara, Japan). The purity and concentration of total RNA were measured and 1 μg of total RNA was reversed transcribed into cDNA using the PrimeScript RT reagent Kit (Takara). The purified SYBR green fluorescent dye mix (Yeasen, China) was used for RT-qPCR analysis in a real-time PCR system (LightCycle 480, Roche) according to the protocols. The relative mRNA levels were calculated by the 2^-ΔΔCt^ method and using primers set were outlined in Table S1.

For RNA-sequencing, total RNA from beige adipocytes treated with control or AZI (5 μM) was isolated with RNeasy mini kit, followed by Rnase Free Dnase treatment (QIAGEN) according to the manufacturer's instructions. mRNA-Seq library preparation was performed using the Illumina TruSeq RNA Library Prep Kit (Illumina). After filtering with an Agilent Bioanalyzer (Agilent), mRNA sequencing was performed on Illumina Hiseq 2500 sequencing system (Illumina) with two terminal sequencing mode and 150-bp paired- end FASTQ read files were generated. Raw mRNA-seq data were trimmed for clean reads, followed by transcript assembly and differential transcripts expression analysis. Top GO software was used for the Gene Ontology (GO) enrichment function analysis while KEGG pathway analysis was annotated and classified in KEGG database.

### Microbiota quantification and 16S rRNA sequencing

The gut microbiota was analysed as previously described 41. For stool bacterial load quantification, total DNA was extracted using the QIAamp Fast DNA Stool Kit (QIAGEN) according to the manufacturer's protocol. The qPCR was performed using primers targeting 16S universal primer (forward: ACTCCTACGGGAGGCAGCAGT; reverse: ATTACCGCGGCTGCTGGC).

For fecal microbiota 16S rRNA sequencing, the region V3-V4 of 16S rRNA genes were amplified and the pooled PCR products were quantified and sequenced using a Novaseq 6000 PE250 (Novogene). The sequences were demultiplexed, quality filtered using the Quantitative Insights into Microbial Ecology (QIIME, version 1.8.0). The QIIME software package was used to conduct the bioinformatic analyses of the sequences. Sequences sharing at least 97% comparability were attributed to the same operational taxonomic units (OTUs). According to the results of species annotation, the top 10 species with the largest abundance in each group at both phylum and family levels were generated to a cylindrical accumulation diagram of species relative abundance. Species composition structure was performed by principal coordinates analysis based on weighted uniFrac distance. Alpha diversity was used to analyze the microbial community diversity within the samples including observed species and Shannon index based on weighted uniFrac distance. Beta diversity was used to examine the sample similarity in the groups based on unweighted unifrac distance.

### Mitochondrial oxygen consumption

Oxygen consumption rate (OCR) was performed using the XF24 Extracellular Flux Analyzers (Seahorse Bioscience) according to the manufacturer instructions. Briefly, primary adipocytes were differentiated for 6 days and treated with or without azithromycin (5 μM) for 24 h. Respiration was measured under basal conditions, following the addition of oligomycin, FCCP, and antimycin A and were normalized by the protein concentrations.

### Human adipose tissue biopsy samples

Human fat biopsies were obtained from the abdominal mesenteric region or subcutaneous fat from 22 obese (BMI ≥ 30 kg/m^2^) and 9 control subjects (18 kg/m^2^ < BMI < 30 kg/m^2^) in the First Affiliated Hospital of Wenzhou Medical University or Ruijin Hospital, Shanghai Jiao Tong University School of Medicine (SJTUSM), with approval of Human Research Ethics Committee of the two hospitals. Written informed consent was obtained from each subject. These subjects were not under AZI treatment for at least a year.

### High performance liquid chromatography-tandem mass spectrometry analysis

The HPLC-MS method was modified according to previous reports 42, 43. Briefly, a minimum of 100 mg tissue is required for each HPLC-MS sample. The pooled samples from human fat biopsy of obese or control subjects or from mice adipose tissues and livers were transferred to the 1.5 mL centrifuge tube and immediately homogenized in 100 μL of phosphate buffer saline (PBS). After suitable sample preparation, the concentration of azithromycin in supernatant was detected by Agilent 1290 HPLC system, coupled with a 6460 triple-quadrupole mass spectrometer and an Agilent Jet Stream electrospray ionization (ESI) source (Agilent Technologies). Total protein from human adipose tissue samples was measured using Enhanced BCA Protein Assay Kit (Thermo Scientific) as per the manufacturer's protocol. The measured azithromycin content was normalized to the total mg amount of protein in the homogenate (ng/mg total protein) for direct comparison between samples.

### Animals

Eight-week-old male C57BL/6J mice were purchased from Shanghai Research Center for Model Organisms and fed according to the guidelines of the East China Normal University Animal Care and Use Committee. Either NCD group (normal chow diet) or HFD group (high-fat diet for 12 weeks, 60% fat) were fed water containing antibiotics as previously reported and the consumption of food and water were record every three days. For acute cold expose, mice were individually caged and exposed to 4 ℃ for 5-6 h for rectal temperature measurement (Braintree) or 7 days for gene analysis. Body fat content was determined every two weeks by AccuFat-1050 NMR (Meg-Med). Whole-body energy expenditure and basal metabolic rate of mice were monitored by a temperature controlled Comprehensive Lab Animal Monitoring System (CLAMS) (Columbus Instruments) for 72 h. Glucose metabolism was measured by glucose tolerance test (GTT) and insulin tolerance test (ITT) in mice treated with or without azithromycin. For GTT analysis, mice were fasted for 16 h and then injected intraperitoneally with glucose in saline solution (1.5 g/kg), and plasma glucose levels were monitored from tail blood at 0, 15, 30, 60, 90, and 120 min (AlphaTrak Blood Glucose Monitor System, Abbott). For ITT analysis, mice were injected intraperitoneally with insulin in saline solution (1.25 U/kg) and plasma glucose levels were monitored at 0, 15, 30, 60, 90, and 120 min.

### Serum parameters and liver triglyceride levels determination

Serum parameters including total cholesterol, high-density lipoprotein cholesterol (HDL-C) and low-density lipoprotein cholesterol (LDL-C) were determined using commercially available assay kits according to the manufacturer's instructions (Jiancheng, China). The lipid was extracted from the liver after being treated with 5% NP-40 solution and heated twice at 90 ℃ and then cooled down to room temperature. The serum and hepatic levels of triglyceride were measured using TG kit (BioVision, USA).

### Histological and immunohistochemistry (IHC) analyses

Tissues (adipose tissues and liver) were dissected and fixed in 10% neutral formalin for 48 h at room temperature. The fixed tissues were dehydrated in ascending series of alcohol, cleared in xylene and embedded in molten paraffin wax. Then paraffin blocks were cut into 5 mm sections and stained with hematoxylin and eosin (H&E). For IHC, sections were deparaffinated and blocked with 5% goat serum for 1 h at RT. The sections were incubated with UCP1 primary antibody (Abcam, England) overnight at 4 ℃ and anti-rabbit HRP-conjugated secondary antibody (Sangon, China) for 1 h at RT. After washing with PBS, the samples were incubated with HRP-DAB followed by nuclear staining with hematoxylin. Sections were examined by light microscopy (Abaton Scan 300/Color scanner).

### Western blotting

Total protein concentrations from cells or tissues were detected using Enhanced BCA Protein Assay Kit (Thermo Scientific). Equivalent amounts of protein were resolved by SDS-PAGE on 10% Tris-glycine gradient gel and transferred to a NC membrane (PALL). Membranes were blocked with 5% nonfat dry milk and incubated overnight at 4 ℃ with the following primary antibodies, including anti-UCP1 (Abcam), anti-PGC1α (Santa Biotechnology, USA), anti-OXPHOS (Abcam) and anti-β-actin (Santa Biotechnology). After washing with PBST (1 L PBS with 500 μl Tween-20) for three times, membranes were incubated for 2 h at RT in the corresponding HRP-conjugated secondary antibodies. After washing in TBST, detection was performed using Odyssey ®CLx Imaging System (LI-COR).

### Detection of intracellular ROS

ROS production in brown and beige adipocytes with different treatments was measured using Reactive Oxygen Species Assay Kit (Beyotime). Briefly, the cells were incubated with DCFH-DA at 37 ℃ for 30 min and then washed with PBS for three times. The fluorescence intensity of DCF was measured using a fluorescent enzyme reader (BMG Labtech) at an excitation wavelength of 488 nm and at an emission wavelength of 525 nm.

### Assay for lipid peroxidation

MDA (malondialdehyde) content was detected using Lipid Peroxidation MDA Assay Kit (Beyotime). Briefly, tissues or cells were homogenized in cell lysis buffer (Beyotime) at 4 ℃. After centrifuging, the supernatant was mixed with thiobarbituric acid (TBA) working solution, and then incubated at 100 ℃ for 15 min. After cooling, sample absorbance was measured at 532 nm using the SpectraMax 190 microplate reader (Molecular Devices).

### Fluorescence and flow-cytometry analysis with mitotracker

Brown and beige adipocytes were incubated with MitoTracker Red CMXRos staining solution (Invitrogen) for 30 min at 37 ℃. Subsequently, the dye solution was replaced by culture medium and fluorescence photograph were taken under a microscope (Nikon). For flow cytometry analysis, the MitoTracker Red treated cells were harvested and resuspended with PBS. Then, the cells were quantitatively detected by a flow cytometer (BD LSRFortessaTM) after filtration and the mean fluorescence intensity of adipocytes was analyzed by FlowJo software.

### Mitochondrial membrane potential analyses

Mitochondrial membrane potential was examined in live cells by mitochondrial membrane potential assay kit with JC-1 (Beyotime). Briefly, cells were washed twice with PBS and incubating with JC-1 working solution for 20 min at 37 ℃ in the dark. After incubation, the relative green (JC-1 monomer) and red fluorescence (JC-1 aggregates) in mitochondria were observed by fluorescence microscopy (Nikon). The ratio of red and green fluorescence was measured using a fluorescent enzyme reader (BMG Labtech).

### Mitochondrial permeability transition pore (mPTP) analyses

mPTP (mitochondrial permeability transition pores) was detected using a mitochondrial permeability transition pore assay kit (Beyotime). Briefly, brown and beige adipocytes were washed twice with PBS and loaded with calcein-AM for 15 min at 37 ℃, following by additional incubation with 1 mM CoCl_2_ for 1 h. After washing with PBS, cells were imaged on a confocal microscope with an argon laser at 488 nm and an emission filter at 525 nm (Leica).

### Cellular ATP measurements

Cellular ATP levels were determined using an Enhanced ATP assay kit (Beyotime). Briefly, lysed cells were centrifuged at 4 ℃ 12000 g for 5 min and the supernatant was transferred into 96-well plate. Then ATP working solution was added per well and incubated for 5 min. Total ATP levels were calculated from the luminescence signals with a luminometer (BMG Labtech).

### Statistical analyses

Statistical analysis was performed using software GraphPad Prism 8. All values specified in figures were represented as mean ± SEM. The two-tailed Student's t test was used for single comparisons. The statistical differences for multiple comparisons between groups were assessed by One-way ANOVA followed by the Dunnett post hoc test. Correlation analysis was calculated using the Spearman's method. The *P*-values were designed as follows: *P < 0.05; **P < 0.01.

## Results

### Azithromycin Suppresses Beige and Brown Adipocytes Gene Program and Functionality

**Figure 1:**
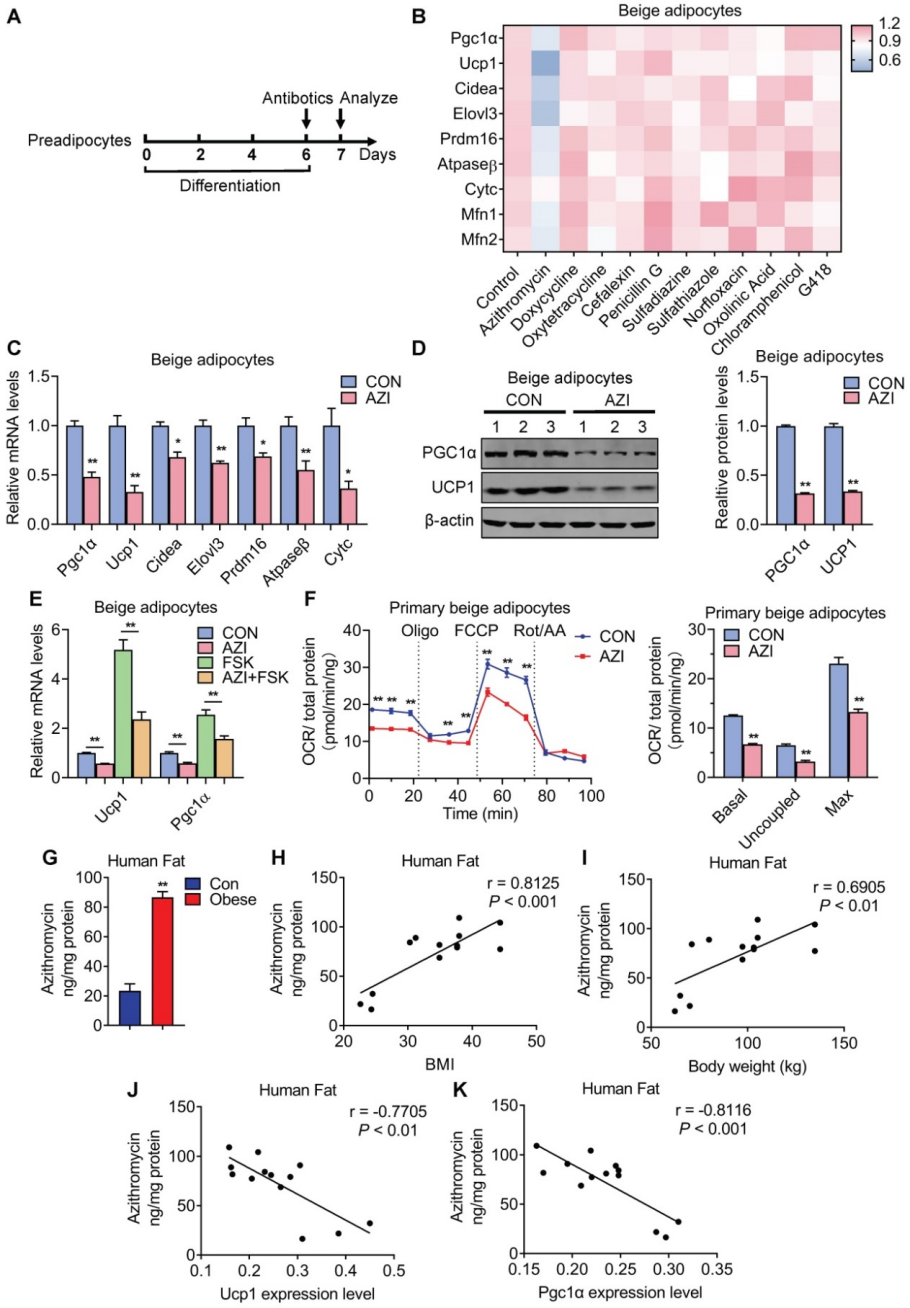
** Antibiotics suppressed brown gene programs in immortal beige adipocytes and its accumulation in adipose tissue of obese patients.** (A) Experimental model of antibiotic treatment in immortal brown and beige adipocytes. (Cells were treated with antibiotics on the sixth day of differentiation and analyzed after treatment for 24 h). (B) Heat map showing thermogenic and mitochondrial gene programs in immortal beige adipocytes treated with different antibiotics at 5 μM for 24 h (n = 3). (C, D) mRNA levels of thermogenic and mitochondrial gene programs (C) and UCP1 and PGC1α protein levels (D) in immortal beige adipocytes treated with or without azithromycin (AZI) at 5 μM for 24 and 48 h (n = 3). (E) mRNA levels of Ucp1 and Pgc1α in immortal beige adipocytes treated with or without AZI at 5 μM for 24 h and forskolin (FSK, 10 μM) for 6 h (n = 3). (F) Oxygen consumption rate (OCR) of primary beige adipocytes treated with or without AZI at 5 μM for 24 h (n = 5). (G) AZI accumulation (ng/mg protein) in sample pools of adipose tissue of control (BMI < 30) or obese (BMI > 30) patients (Con n = 3; Obese n = 10). (H-K) Correlation between AZI levels and BMI (H), body weight (I), Ucp1 (J) and Pgc1α mRNA levels (K) (n = 13). Data are presented as mean ± S.E.M. and *P < 0.05, **P < 0.01 compared to control group.

In order to clarify the direct influences of antibiotics on brown and beige adipocyte, we screened major types of antibiotics commonly used in clinic, on livestock and in aquaculture 44-46, including Azithromycin (macrolides), Doxycycline and Oxytetracycline (tetracyclines), Cefalexin and Penicillin G (beta-lactams), Sulfadiazine and Sulfathiazole (sulfonamides), Norfloxacin (quinolones), Oxolinic Acid (quinoline ketone), Chloramphenicol (amide alcohol) and G418 (aminoglycosides) to examine their effects on the differentiation capacity and thermogenic functionality of immortal brown and beige adipocytes (Figure 1A). Oil red staining and quantification analysis showed that these antibiotics have minor effects on adipocyte differentiation, which was confirmed by marginal differences in adipogenesis marker PPARγ expression upon antibiotics treatment compared to individual controls (Figure S1A-D). Interestingly, compared to other antibiotics in the screen panel, we found that azithromycin (AZI) specifically and effectively suppressed the mRNA levels of thermogenic (Pgc1α, Ucp1, Cidea, Elovl3 and Prdm16) and mitochondrial gene programs (Atpaseβ, CytC, Mfn1 and Mfn2) in immortal brown and beige adipocytes, without affecting cell viability (Figure 1B and Figure S1E-F). Interestingly, we found that among different generations of macrolides (Erythromycin, Roxithromycin and Telithromycin), AZI exhibited the most consistent and potent effects on thermogenic and mitochondrial functionality inhibition on immortal brown and beige adipocytes (Figure S1G). Furthermore, *in vivo* screens using inguinal fat samples from mice injected unilaterally in iWAT with control or various antibiotics rendered consistent results as *in vitro* screens (Figure S2A-B). Intravenous delivery of AZI in mice also confirmed the screen results, which showed suppressed brown gene programs in iWAT compared to control (Figure S2C). These data suggest that AZI may be a potent suppressor of brown and beige adipocytes functions.

Indeed, further analysis showed that AZI significantly suppressed thermogenic and mitochondrial gene programs as well as UCP1 and PGC1α protein levels in both immortal beige and brown adipocytes (Figure 1C-D and Figure S3A-B). Moreover, AZI treatment largely blocked thermogenic activation induced by forskolin, a well-established cAMP agonist that promotes beige/brown adipocyte heat production in immortal beige adipocytes 47 (Figure 1E and Figure S3C). We further used Seahorse flux analyzer to assess oxygen consumption rate (OCR) for the reflection of mitochondrial respiratory and thermogenic capacity of adipocytes. Of note, compared to control group, AZI-treated primary beige adipocytes exhibited a significant reduction in basal respiration, proton leak and maximal respiratory capacity (Figure 1F). We observed similar repression, though to a lesser extent, in OCR levels in primary brown adipocytes upon AZI administration (Figure S3D). Together, these results suggest that AZI treatment impaired beige/brown adipocyte functionality.

### Enhanced Azithromycin accumulation in adipose tissue of obese patients that correlates with BMI

Next, we examined whether there is AZI accretion in adipose tissues and whether its levels correlate with metabolic status in humans. Of clinical and therapeutic significance, we found increased AZI accumulation in adipose tissues from obese subjects compared to control subjects (Figure 1G). Moreover, AZI levels in adipose tissues correlated positively with Body Mass Index (BMI) and body weights in humans, while inversely correlated with mRNA levels of Ucp1 and Pgc1α in human adipose tissues (Figure 1H-K). Overall, these data suggested that there is significant AZI accumulation in human fat tissues, with no difference between abdominal mesenteric region or subcutaneous fat (Figure S3E), possibly stemmed from environmental pollutants, and that this accumulation may play an instigating role in obesity development.

### Azithromycin exposure leads to increased adiposity and metabolic dysfunctions in mice under high fat diet

**Figure 2:**
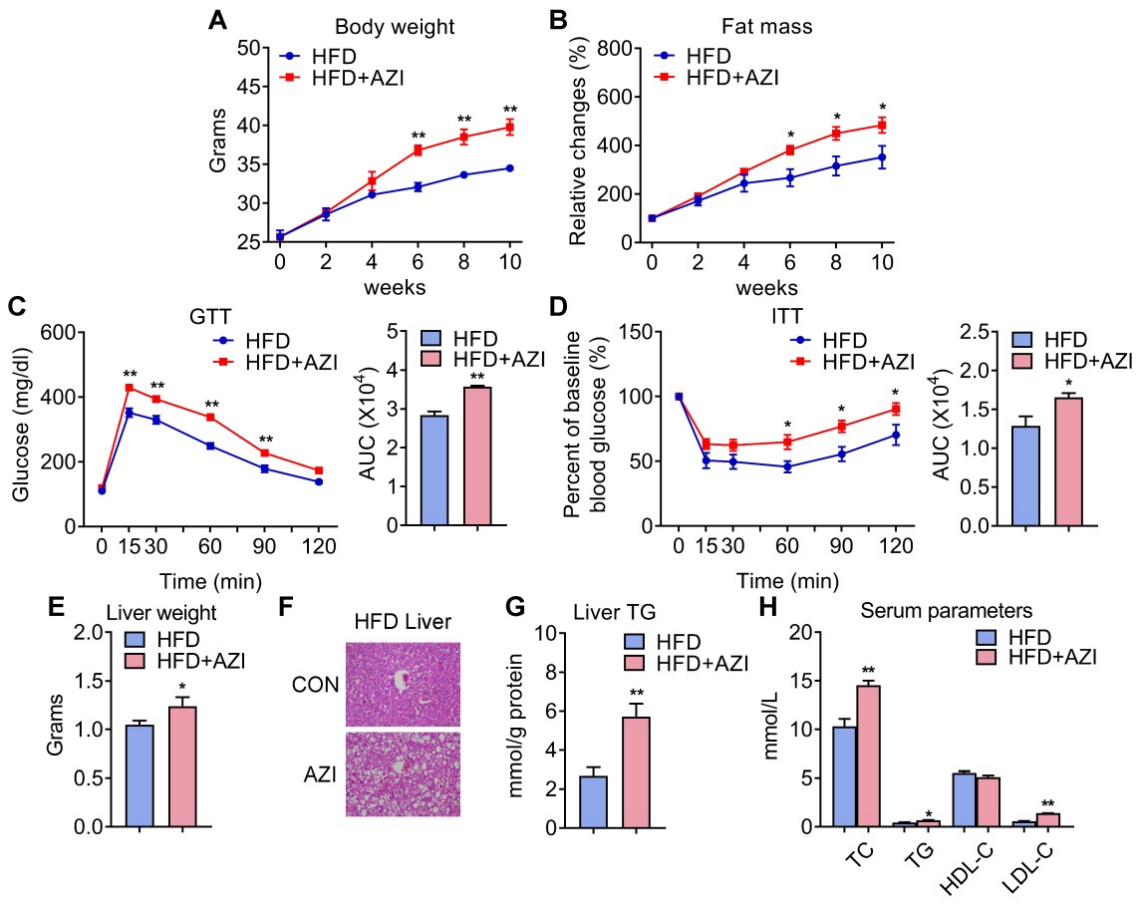
** Azithromycin treatment induced adiposity and metabolic dysfunctions in mice for HFD.** (A-H) Metabolic performances of HFD fed mice treated with or without AZI via drinking water for 12 weeks. (ABX is given throughout the whole process) (ABX: 1.0 g/L; AZI: 50 mg/kg/day) (n = 6 per group). (A) Body weight; (B) Relative change of fat mass; (C) Glucose tolerance test (GTT) and area under the curve (AUC) analysis; (D) Insulin tolerance test (ITT) and AUC analysis; (E) Liver weight, (F) Representative H&E staining of liver; (G) Liver triglycerides levels and (H) Serum parameters. Data are presented as mean ± S.E.M. and *P < 0.05, **P < 0.01 compared to control group.

Since AZI treatment impaired brown and beige adipocytes functionality *in vitro* and its levels are elevated in fat tissues of obese patients, we then set out to unravel the effects and mechanisms of AZI exposure in mice. Mice were randomly assigned to control group, or AZI group with AZI supplemented in drinking water at a previously reported safe dosage 38. Of note, antibiotics have been shown to impact obesity onset by interfering with intestinal flora. Thus, we treated mice with ampicillin and neomycin (ABX) from seven days prior to AZI treatment and maintained throughout the whole experimental process to circumvent the possible influences of gut microbiota on data interpretation. Under normal chow diet (NCD), mice with AZI treatment (NCD+AZI) showed similar metabolic performances compared to control mice (NCD) (Figure S4A-G). Strikingly, under high fat diet (HFD), we observed increased body weights and fat mass, damaged insulin sensitivity as shown by worsen performances in glucose and insulin tolerance tests (GTT/ITT) in AZI group (HFD+AZI) as compared to control group (HFD) (Figure 2A-D). Meanwhile, AZI-treated mice characterized increased extent of liver steatosis under HFD as shown by increased liver weights, lipid infiltration and intrahepatic triglycerides levels, while serum analysis showed increased serum total triglyceride, total cholesterol and low-density lipoprotein-cholesterol levels compared to controls (Figure 2E-H). Taken together, these data suggest that AZI exposure aggravates obesity and metabolic dysfunctions in mice under HFD challenge.

### Azithromycin exposure inhibited BAT and iWAT functionality in mice

**Figure 3:**
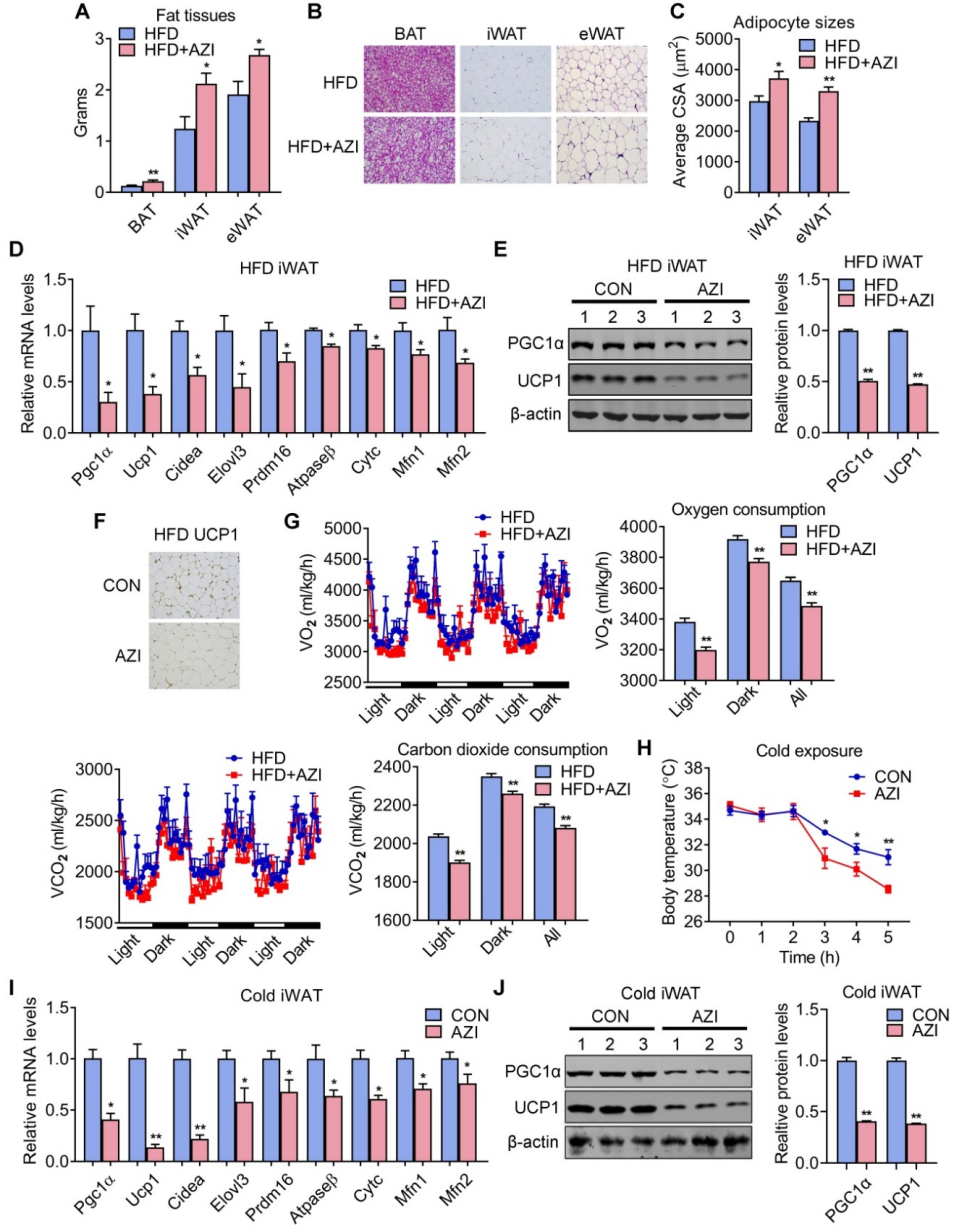
** Azithromycin treatment suppressed energy expenditure and thermogenic capacity in mice for HFD.** (A-G) Metabolic performances of HFD fed mice treated with or without AZI via drinking water for 12 weeks (ABX is given throughout the whole process) (ABX: 1.0 g/L; AZI: 50 mg/kg/day) (n = 6). (A) Tissue weights of brown (BAT), inguinal (iWAT) and epididymal (eWAT) fat pads. (B, C) Representative images of H&E staining of fat tissues (B) and quantitative analysis of adipocyte sizes (C) of iWAT and eWAT. (D-F) mRNA levels of thermogenic and mitochondrial gene programs (D); Western blot and quantitative analysis of UCP1 and PGC1α protein levels (E); Representative images of UCP1 immunostaining (F) in iWAT. (G) Energy expenditure was determined as oxygen consumption and carbon dioxide consumption. (H-J) Rectal temperatures of mice during 5 h cold exposure (H), as well as mRNA levels of thermogenic and mitochondrial gene programs (I) and representative UCP1 and PGC1α protein levels (J) in iWAT of mice after 5 h cold exposure (n = 6). Data are presented as mean ± S.E.M. and *P < 0.05, **P < 0.01 compared to control group.

We next investigated the intrinsic mechanism of enhanced obesity and metabolic derangement in AZI-treated mice under HFD. We first evaluated the influence of AZI treatment in terms of AZI residue in an array of adipose and liver tissues. Interestingly, the results revealed significant AZI remnant in adipose tissues upon treatment, as well as higher AZI remnant in liver, an organ considered as a central hub handling exogenous elements, indicating a predisposition of fat tissues to antibiotics residue (Figure S5A). Of note, the concentration of AZI in mice adipose tissues were at the similar extent compared to that in obese patients (Figure S5A and Figure 1G). Next, anatomic analysis revealed that AZI-treated HFD mice featured increased adipose tissue weights with enlarged adipocyte sizes as shown by H&E staining and cross-sectional area (CSA) quantification (Figure 3A-C). The mRNA levels of thermogenic and mitochondrial gene programs as well as PGC1α and UCP1 protein levels were significantly suppressed, while inflammatory gene expressions were increased, in iWAT and BAT of AZI-treated HFD mice, indicating the thermogenic functions in these mice were disrupted, while exhibited mild or no effects on mitochondrial gene programs in liver and gastrocnemius muscle (Figure 3D-F and Figure S5B-E). Indeed, we observed a consistent decrease in energy expenditure levels in AZI-treated mice compared to controls, as demonstrated by reduced whole body oxygen and carbon dioxide consumption in AZI group, while locomotor activity and food intake remained unaltered between two groups (Figure 3G and Figure S5F). Furthermore, as cold-induced thermogenesis in iWAT and BAT is key for body temperature defense in cold environment 48, we found that AZI mice showed impaired thermogenic capacity as shown by their accelerated drop in core temperature during a cold challenge (Figure 3H). Subsequent analysis revealed that AZI mice had blunted expression of thermogenic and mitochondrial gene programs as well as decreased PGC1α and UCP1 protein levels, compared to controls after cold challenge (Figure 3I-J and Figure S5G-H).

Taken together, these results suggested that AZI exposure promotes adiposity in mice under HFD by impairing browning of white fat and energy expenditure.

### Azithromycin causes mitochondrial dysfunction and oxidative damage in brown and beige adipocytes

AZI has been reported to affect mTOR signaling and the autophagy process, which may potentially contribute to the development of obesity 49-51. However, we found comparable protein content, as well as phosphorylated mTOR levels in immortal brown and beige adipocytes with or without AZI treatment (Figure S6A-D). Meanwhile, AZI exposure failed to cause significant changes in the expression of two key autophagy marker LC3I and LC3II in immortal brown and beige adipocytes (Figure S6E-F), suggesting AZI may impair thermogenic function of iWAT and BAT by means other than its influence of mTOR signaling or autophagy.

**Figure 4:**
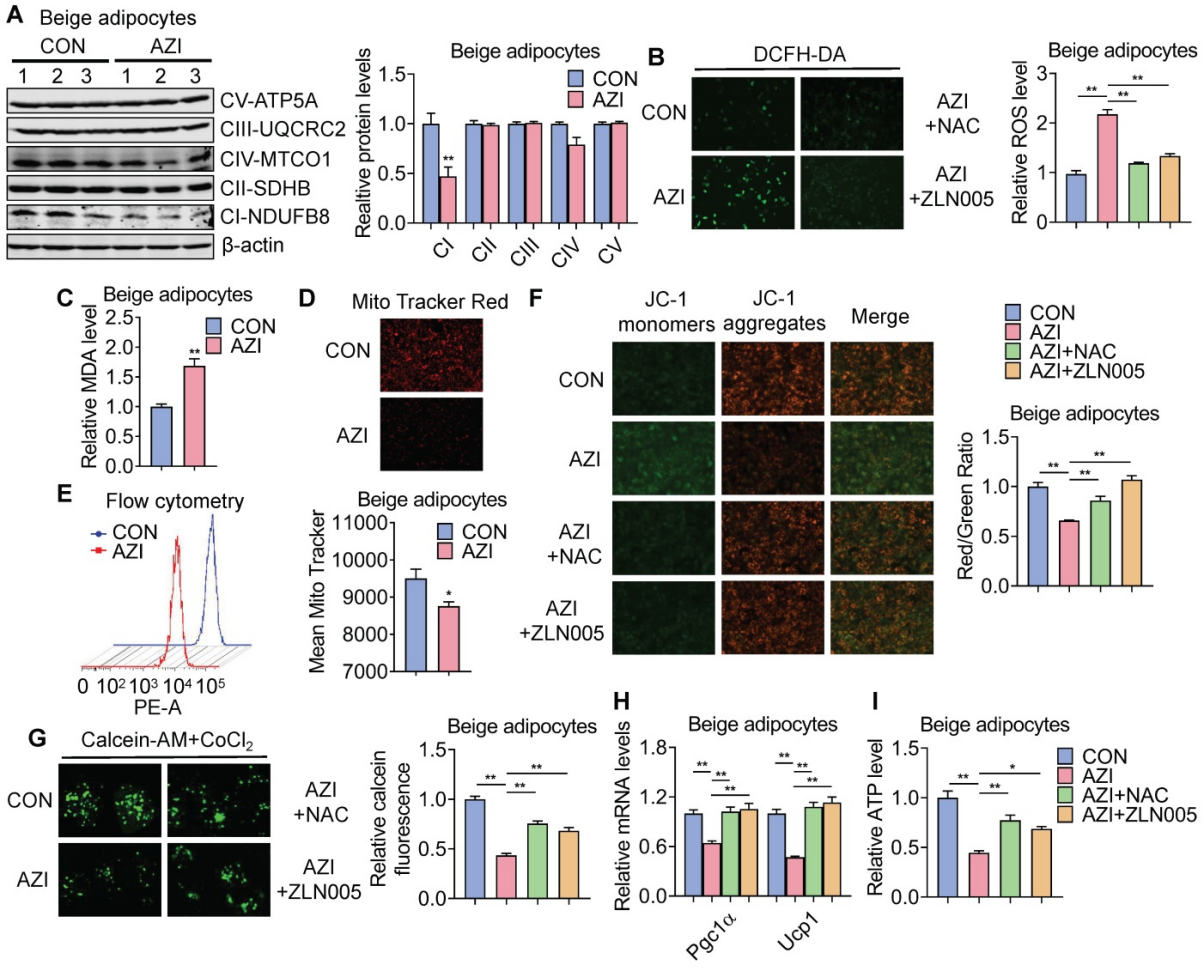
** Azithromycin induced mitochondrial dysfunction and oxidative damage in immortal beige adipocytes.** (A) Protein levels of major mitochondrial OXPHOS components in immortal beige adipocytes treated with or without AZI (n = 3). (B-I) Immortal beige adipocytes treated with control (CON), AZI (5 μM), AZI (5 μM)+N-Acetylcysteine (NAC, 5 mM) or AZI+ZLN005 (10 μM) (n = 3). (B) Reactive oxygen species (ROS) staining and quantification; (C) Relative malondialdehyde (MDA) levels; (D, E) Mitotracker fluorescent staining and flow cytometry analysis of fluorescence intensity; (F) Mitochondrial membrane potential by JC-1 staining analysis; (G) Mitochondrial Permeability Transition Pore (mPTP) analysis; (H) Ucp1 and Pgc1α mRNA levels and (I) Relative ATP levels (n = 3). Data are presented as mean ± S.E.M. and *P < 0.05, **P < 0.01 compared to control group.

We thus performed RNA-seq to understand the global changes of gene expressions upon AZI treatment on immortal beige adipocytes. Of note, KEGG pathway and GO enrichment analysis consistently showed that oxidative phosphorylation and mitochondrial functionalities were mostly affected upon AZI treatment in beige adipocytes (Figure S6G-H). Notably, as a Macrolide antibiotic, AZI exerts its anti-bacteria properties via targeting bacterial ribosomes and rRNA, which hindered ribosome assembly and inhibited bacterial protein synthesis 52, 53. With its bacterial origin, mitochondrial ribosomes share structural similarity with their counterparts in bacteria ribosomes, thus may inhibit protein synthesis of mitochondrial components and rendering mitochondrial susceptible to AZI exposure. Consistent with this idea, we found that protein levels of mitochondrial electron transport chain (ETC) component I was consistently reduced in both immortal brown and beige adipocytes exposed to AZI in *in vitro* and *in vivo* models, as well as significantly reduced component III in iWAT (Figure 4A and Figure S7A-B).

ETC defects have been demonstrated to increase mitochondrial reactive oxygen species (ROS) production and cause oxidative damage to cells 54, 55. Indeed, compared to controls, we found increased ROS levels in AZI-treated immortal brown and beige adipocytes (Figure 4B and Figure S7C) as shown by elevated ROS staining and enhanced levels of malondialdehyde (MDA), an end product of lipid peroxidation induced by ROS over-production 56 (Figure 4C and Figure S7D). The perturbation in ROS levels under AZI treatment in turn disrupted mitochondrial membrane potential as demonstrated by decreased fluorescence signals in MitoTracker staining under both microscopy and flow cytometry examination, as well as increased monomers and decreased aggregates signaling under JC-1 staining (Figure 4D-F and Figure S7E-G). Moreover, AZI treatment impaired mitochondrial functionality of adipocytes as shown by enhanced opening of mitochondrial permeability transition pore (mPTP), decreased Ucp1 and Pgc1α mRNA levels, and reduced production of adenosine triphosphate (ATP) (Figure 4G-I and Figure S7H-J).

Of note, treating immortal adipocytes with anti-oxidant N-Acetylcysteine (NAC) to clear excessive ROS, or with Pgc1α activator ZLN005 57, 58 to enhance mitochondrial biogenesis largely corrected the defects in ROS levels, mitochondrial membrane potential with decreased monomers and increased aggregates under JC-1 staining, impeded mPTP opening, while at the same time rescued Pgc1α and Ucp1 expression levels and ATP production (Figure 4B and F-I and Figure S7C and G-J). Moreover, we found that NAC at 5 mM did not severely affected acidification of culture medium, thus may not give rise to secondary effects on mitochondrial function *in vitro* (Figure S7K).

Taken together, these data suggest that AZI inhibited protein synthesis of mitochondrial major components, which produced ROS and disrupted mitochondrial functionality in immortal brown and beige adipocytes. Importantly, anti-oxidant NAC or Pgc1α activator ZLN005 supplement alleviates azithromycin exposure induced mitochondrial dysfunctions in immortal brown and beige adipocytes.

### Anti-oxidant N-Acetylcysteine (NAC) supplement alleviates azithromycin exposure induced obesity and metabolic dysfunctions in mice under high fat diet

**Figure 5:**
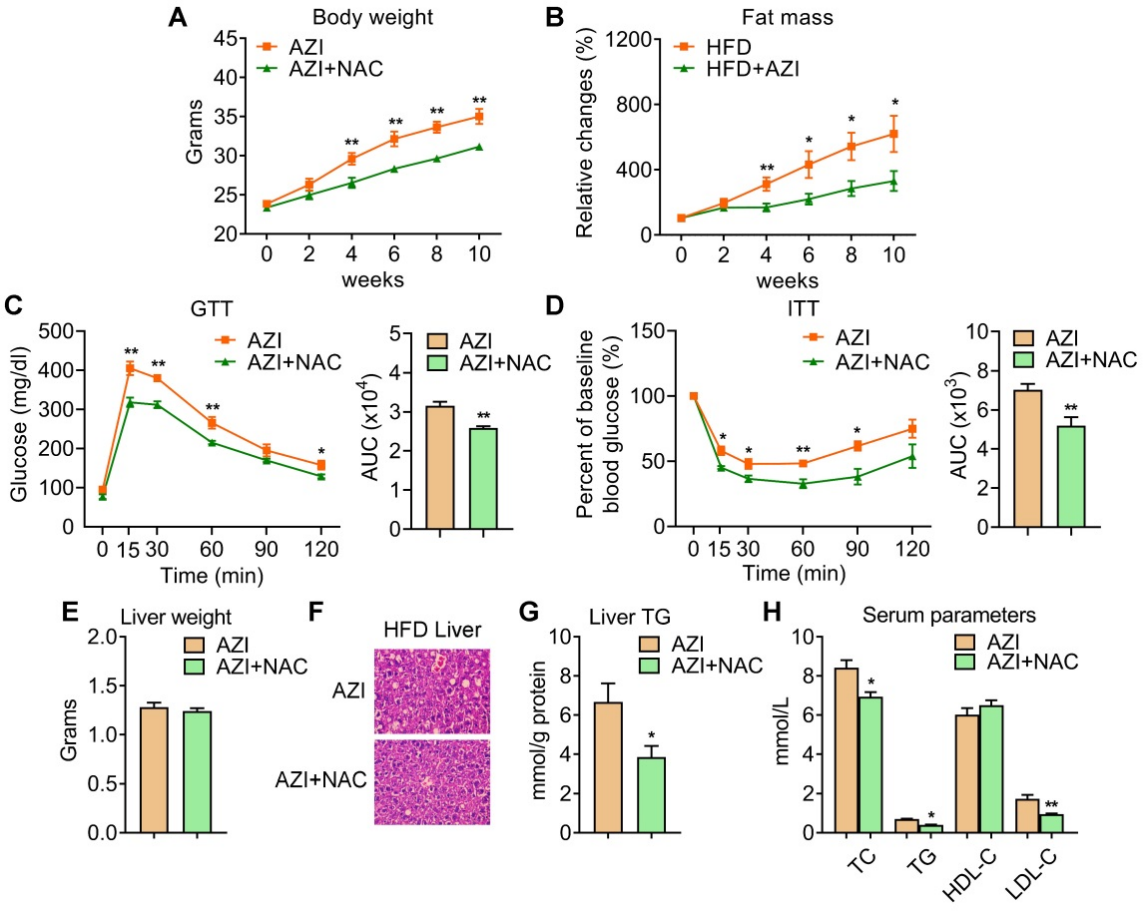
** Anti-oxidant N-Acetylcysteine (NAC) supplement alleviated azithromycin exposure induced obesity and metabolic dysfunctions in HFD mice.** (A-H) Metabolic performances of HFD fed mice treated with AZI or AZI+NAC via drinking water for 12 weeks. (ABX is given throughout the whole process, ABX: 1.0 g/L, AZI: 50 mg/kg/day and NAC:1.5 g/kg/day, n = 6). (A) Body weight; (B) Relative change of fat mass; (C) Glucose tolerance (GTT) and area under the curve (AUC) analysis; (D) Insulin tolerance test (ITT) and AUC analysis; (E) Liver weight, (F) Representative H&E staining of liver; (G) Liver triglycerides levels and (H) Serum parameters. Data are presented as mean ± S.E.M. and *P < 0.05, **P < 0.01 compared to control group.

We have demonstrated that AZI suppressed mitochondrial function through disruption of ETC components and ROS production. Importantly, treating immortal adipocytes with anti-oxidant N-Acetylcysteine (NAC) is sufficient to reinstall cellular mitochondrial functionality (Figure 4 and Figure S7). We then explored whether NAC supplement would alleviate the deleterious effects of AZI on metabolic parameters *in vivo*. To test this, mice were first treated with ABX before and throughout the whole experimental process to eliminate the influence of gut microbiota on obesity development, then subjected to a HFD regime and treated with AZI, with or without NAC supplement. Of note, NAC supplement in AZI-treated HFD mice largely prevented AZI-induced gain of body weight and fat mass (Figure 5A-B). Compared to controls (AZI), mice treated with NAC (AZI+NAC) showed improved metabolic performances, as demonstrated by better glycemic control in GTT and ITT, decreased extent of liver steatosis and improved serum lipid profile (Figure 5C-H).

**Figure 6:**
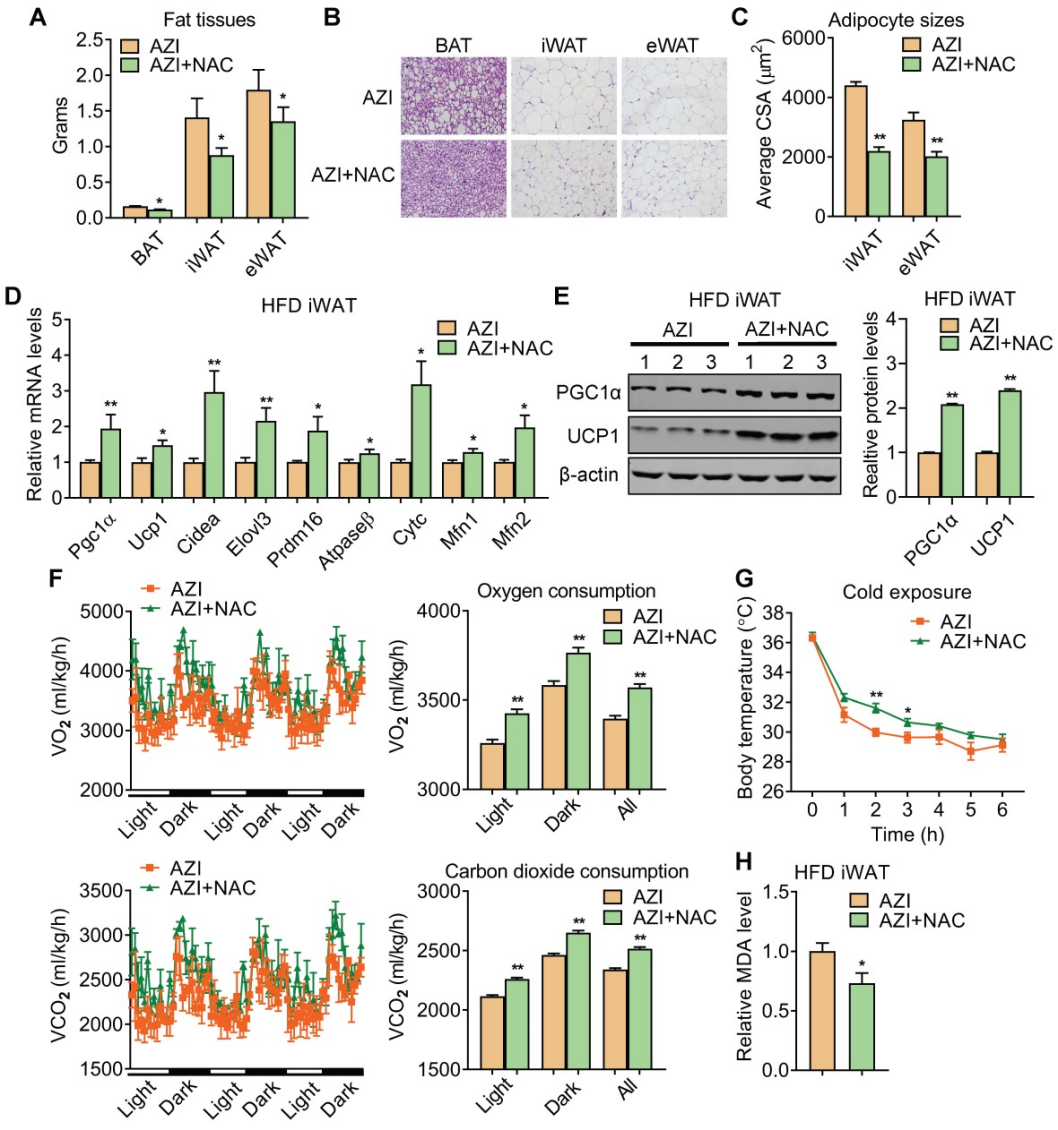
** Anti-oxidant N-Acetylcysteine (NAC) supplement restored energy expenditure and thermogenic capacity in azithromycin treated mice under HFD.** (A-H) Metabolic performances of HFD fed mice treated with AZI or AZI+NAC via drinking water for 12 weeks (ABX is given throughout the whole process, ABX: 1.0 g/L, AZI: 50 mg/kg/day and NAC:1.5 g/kg/day, n = 6). (A) Tissue weights of brown (BAT), inguinal (iWAT) and epididymal (eWAT) fat pads. (B, C) Representative images of H&E staining of fat tissues (B) and quantitative analysis of adipocyte sizes (C) of iWAT and eWAT. (D, E) mRNA levels of thermogenic and mitochondrial gene programs (D) and UCP1 and PGC1α protein levels (E). (F) Energy expenditure was determined as oxygen consumption and carbon dioxide consumption. (G) Rectal temperatures of mice during 6 h cold exposure. (H) Relative malondialdehyde (MDA) levels in iWAT of mice.Data are presented as mean ± S.E.M. and *P < 0.05, **P < 0.01 compared to control group.

Detailed analysis showed that NAC supplement in mice significantly reduced adipose tissue weights (Figure 6A) with smaller adipocyte sizes (Figure 6B-C). This was accompanied with an overall restoration in expressions of thermogenic and mitochondrial gene programs and protein levels of UCP1 and PGC1α, whole-body oxygen and carbon dioxide consumption and thermogenic capacity under cold stimulation in iWAT and BAT of AZI+NAC treated HFD mice compared to AZI-treated HFD mice, without obvious changes in locomotor activity and food intake between two groups (Figure 6D-G and Figure S8A-C). Furthermore, NAC administration also decreased MDA levels in mice, indicating a reduction in lipid peroxidation and an improvement in mitochondrial ROS balance (Figure 6H). Taken together, these data suggest that antioxidant NAC alleviates AZI exposure-induced obesity and metabolic dysfunctions in mice under HFD, at least by restoring ROS balance in mitochondrial of adipocytes.

Of note, in order to decipher the contribution of adipose tissues and microbiota to the metabolic changes of mice, we examined the microbiota quantity in mice feces after ABX pretreatment and found that ABX administration successfully depleted microbiota, which is consistent with previous reports 59, 60. We further performed 16S rRNA sequencing to examine residual resistant microbiota composition after 12 week intervention. Interestingly, we found that Firmicutes and Proteobacteria were the most abundant resistant microbiota at phylum level, in which the dominant bacteria were Streptococcaceae and Pseudomonadaceae at family level, respectively. While, no significant differences in microbiota composition were observed among ABX, ABX+AZI and ABX+AZI+NAC groups, as shown by PCoA plot and Alpha/Beta diversity analysis (Figure S9 and Table S2).

In addition, we also deliver CON, AZI and AZI+NAC in HFD fed mice via chronic i.p. injection to bypass the influences of microbiota. Consistent with the data shown with AZI and NAC administration via water drinking, i.p. injection of AZI in mice also showed significantly impaired metabolic performances, including increased adiposity, insulin resistance, impaired thermogenic capacity, reduced energy expenditure and hepatic steatosis, as well as suppressed thermogenic and mitochondrial gene programs and increased inflammatory gene levels in iWAT and BAT compared to control mice, while NAC treatment reversed the adverse effects of AZI treatment via i.p. delivery (Figure S10 , Fgure S11 and Figure S12). Overall, these data suggested that AZI exposure induced obesity and metabolic dysfunctions in mice under high fat diet and antioxidants improved the phenotype majorly via adipose tissues, instead of microbiota.

## Discussion

In recent decades, obesity has become a global epidemic threatening public health. Great research efforts have been made to elucidate the pathogenesis of obesity and metabolic syndrome 2. Epidemiological studies indicated that, besides the prevalence of modern life style featuring minimal physical activity and excessive energy intake, exposures to diversified environmental pollution also play a prominent role in the etiology of obesity 61. In the present study, we have found that azithromycin (AZI), one major kind of macrolide antibiotics, specifically disrupted thermogenic and mitochondrial functions in brown and beige adipocytes via enhancement of cellular ROS levels and led to obesity in mice and in human. Considering the wide use, even abuse, of AZI in various aspects of human life, i.e. in clinic, animal husbandry, aquaculture, our study suggested a possible role of AZI in obesity epidemics, and offered proof-of-concept evidences for cautious antibiotics applications in the future.

In this study, we collected fat biopsies from obese and lean human subjects to analyze the AZI accumulation. One limitation of this analysis is that due to clinic ethics, the quantities of fat biopsy taken from each subject were limited and several fat samples were pooled for AZI examination because of the large sample volume requirement for fat tissues in mass spectrometry analysis. Nevertheless, it is intriguing that, using fat biopsies from humans, we found increased AZI accumulation in adipose tissues of obese versus control subjects, as well as a positive correlation between fat AZI concentration and human BMI/body weight, thus strongly suggested the involvement of AZI in human obesity. Further studies to examine brown/beige fat activation in human via 18F-FDG PET-CT and correlate the data with AZI concentration in human brown fat biopsy would be more helpful to establish a direct correlation. Of note, considering that the half-life of most antibiotics is in a range of several hours to no longer than one week 62, 63 and the participates were not under AZI treatment for at least a year, it is probable that the AZI accretions in human fat are originated from environmental antibiotics pollution, for example, daily consumption of drinking water, livestock or fishes that having AZI residues. Using AZI as an example, our results signified the importance of establishing further guidelines for the use and the residue monitor of antibiotics in water treatment, animal husbandry and aquaculture industry, etc. in order to prevent possible environmental antibiotics pollution.

Another interesting finding is that we found AZI exposure specifically affected metabolic performances in obese mice under HFD but not in lean mice under chow diet via drinking water. It has been shown that lipid overload may promote ROS generation in adipocytes and in adipose tissues 64. Thus, AZI may exacerbate oxidative stress caused by lipid toxicity in the scenario of over nutrition. Food of high energy density is easy to obtain in modern society and have great popularity for their deliciousness originated from the high fat/sugar contents. It is thus possible that this feature of the modern world, combining with the enriched environmental antibiotic pollutions, i.e. AZI, have made people prone to develop metabolic derangement. Nevertheless, acute delivery of AZI in iWAT or tail vein led to suppressed brown gene program in iWAT of NCD mice, suggesting the complexity of AZI influences in adipose tissues, which needs further investigations.

Interestingly, we noticed that brown fat seems less sensitive to AZI under cold exposure compared to beige fat, suggesting the tissue- and cellular-specific effects upon AZI exposure. The different responses of beige and brown adipocytes to metabolic stresses were frequently reported. For example, both PRDM16 and PGC-1α are well-known master regulators for energy homeostasis. Of note, fat-specific PGC-1α deficient mice developed insulin resistance and hyperlipidemia and the effects were majorly caused by beige fat as shown by decreased expression of thermogenic and mitochondrial genes, whereas gene expression patterns in brown fat were not altered 65. In addition, mice of adipocyte-specific PRDM16 deletion had inhibited beige adipocyte function in subcutaneous fat and developed obesity, as well as severe insulin resistance and hepatic steatosis under HFD, while caused minimal effects on brown fat 66. It is possible that AZI may also disrupt other mitochondria enriched metabolic organs, which warrants systematical evaluation of its metabolic influences in the future 67.

Notably, gut microbes have been shown to be critical for obesity development. In this study, germ-free mice may be useful to better decipher whether gut microbiota contributing to the AZI's impacts on obesity and metabolic parameters. However, germ-free mice were reported to have defects in intestinal nutrition absorbance 68, 69, which rendered these mice highly resistant to diet induced obesity 70. Considering our results that AZI promotes obesity under high fat diet feeding while exhibited minor effects under chow diet, we speculated that AZI's effects may not be able to manifest in germ-free mice in the current study. Thus, we in turn co-treated mice with ABX during the whole experiment, which eliminated the majority of gut microbiota. Of note, via 16S rRNA sequencing on the residual flora, we found that ABX, ABX+AZI and ABX+AZI+NAC groups all featured similar microbiome compositions. In addition, we have further performed acute iWAT or i.v. injection of control and AZI in mice to circumvent the influence of microbiota, which resulted in consistent suppression of thermogenic and mitochondrial gene programs in iWAT. Moreover, chronic i.p. administration of AZI in mice (no ABX treatment) also led to similar metabolic deleterious effects as in AZI water-feeding models. Overall, these results suggested that the contribution of microbiota on AZI's metabolic influences was limited in our study. Interestingly, a recent report shows that the quinolone Enoxacin also exhibited direct effects on adipose tissues but not via microbiota 32.

Different categories of antibiotics characterize distinct bactericidal mechanisms based on their unique chemical structures. Majorly, antibiotics eliminating bacterial through four types of mechanisms, including inhibition of bacterial cell wall synthesis, interaction with bacterial cell membrane, interference of bacterial protein synthesis and inhibition of bacterial nucleic acid replication/transcription 71, 72. Previous reports have shown that bactericidal antibiotics, including Ciprofloxacin (quinolone), Ampicillin (β-lactam), and Kanamycin (aminoglycoside), lead to breakdown of tricarboxylic acid (TCA) cycle and electron transport chain (ETC) and consequently increased oxidative stresses in epithelial cells 30. On the other hand, Enoxacin (another quinolone) induced intrinsic oxidative metabolism in a beneficial way, which resulted in enhanced TCA cycle, downregulation of mir-34a-5p and increased FGF21 signals, and eventually increased beige adipogenesis and ameliorated obesity 32. Besides, recent work showed that Doxycycline (tetracyclines) prevented cell death and inflammation in severe mitochondria disease (MD) mutant cells and complex I deficiency (Ndufs4^-/-^) mice featuring Leigh syndrome by inducing mitohormetic response in neuronal system 73. In the present study, we observe minor changes in brown/beige adipocyte functionality upon quinolone (Norfloxacin), β-lactam (Penicillin G) or aminoglycoside (G418) treatment, while macrolide AZI treatment promoted ROS production and obesity by reducing protein levels of mitochondrial electron transport chain (ETC) components I and III. Thus, the results of our study and others overall suggested that the physiological effects of antibiotics on ROS production and on energy metabolism are dependent on the distinct bactericidal mechanisms and on cell types, i.e. epithelial cells, MD mutant cells and adipocytes 30.

In conclusion, our results revealed that AZI exposure disrupts energy homeostasis and promotes adiposity via suppressing brown and beige adipocytes functionality. In detail, AZI decreased mitochondrial protein synthesis and increased ROS levels in thermogenic adipocytes, which could be attenuated by NAC supplement both *in vitro* and *in vivo*. Of note, we found positive correlation of AZI residual levels in human fat and BMI/body weight, emphasizing the importance of AZI in obesity epidemics and warning against the AZI abuse in the future.

## Supplementary Material

Supplementary figures and tables.

## Abbreviations

BMI: Body Mass Index
ROS: Reactive oxygen species
NAC: N-Acetyl-L-cysteine
AZI: Azithromycin
OCR: Oxygen consumption rate
ABX: Ampicillin and neomycin
BAT: Brown adipose tissue
iWAT: Inguinal white adipose tissue
eWAT: Epididymal white adipose tissue
HPLC-MS: High performance liquid chromatography-tandem mass spectrometry
