# Supplementary material for: Antibiotic Azithromycin inhibits brown/beige fat functionality and promotes obesity in human and rodents
Source: Theranostics. 2022 Jan 1;12(3):1187–203. doi: 10.7150/thno.63067 (PMC8771569; doi:10.7150/thno.63067)
Supplement: Supplementary file 1 — Supplementary figures and tables. [file thnov12p1187s1.pdf]

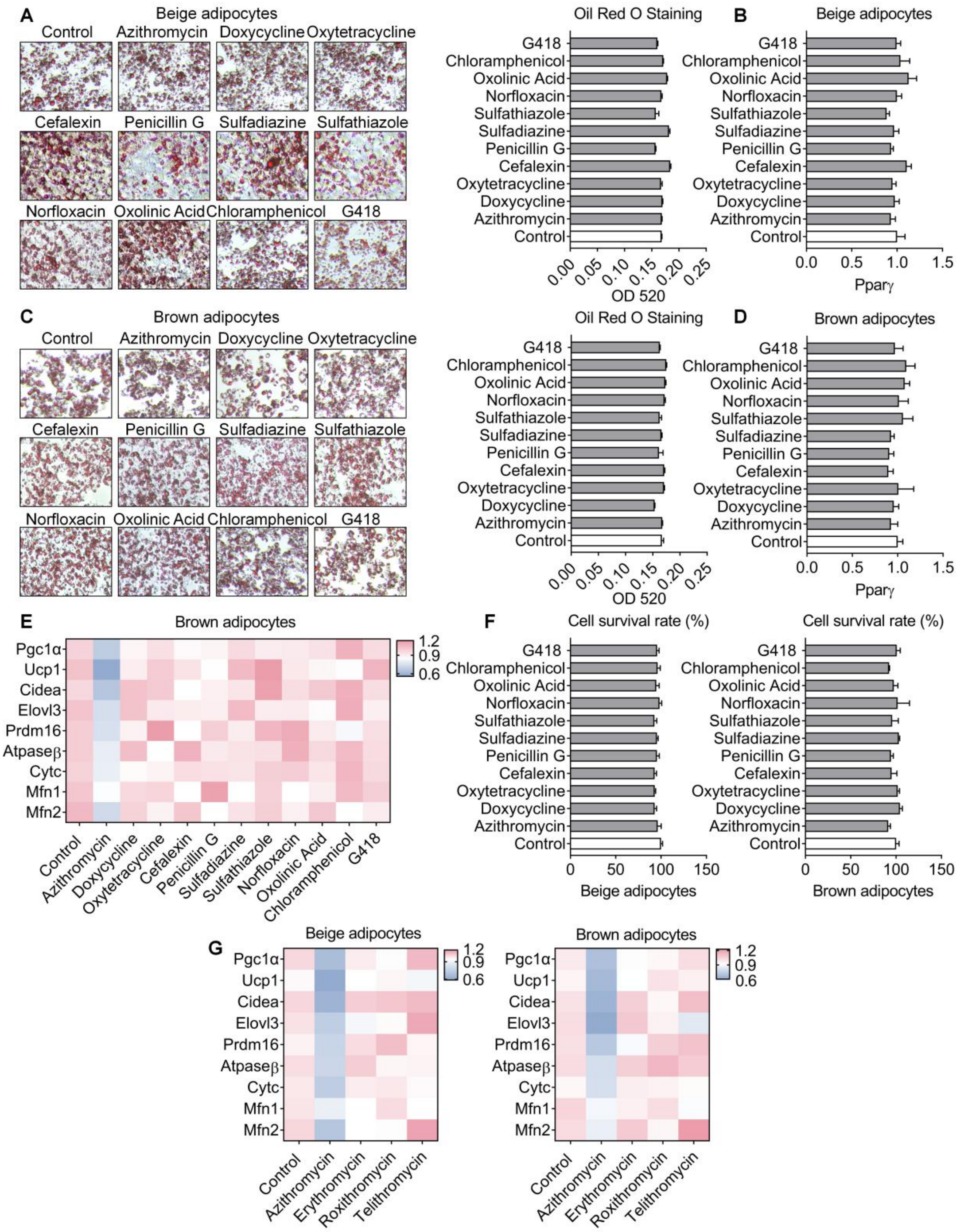

Figure S1. Azithromycin inhibited thermogenic and mitochondrial gene programs of immortal beige and brown adipocytes without affecting cell differentiation and viability

(A-D) Representative images of Oil red staining (A, C) and mRNA levels of Pparγ (B, D) of immortal beige and brown adipocytes treated with or without AZI at 5 μM for 24 h (n = 3).

(E) Heat map showing thermogenic and mitochondrial gene programs in immortal

brown adipocytes treated with different antibiotics at 5 μM for 24 h (n = 3).

(F) Cell survival rate of immortal beige and brown adipocytes treated with different antibiotics at 5 μM for 24 h (n = 3).

(G) mRNA levels of thermogenic and mitochondrial gene programs in immortal beige and brown adipocytes treated with controls, erythromycin, azithromycin, roxithromycin and telithromyc at 5 μM for 24 h (n = 3).

Data are presented as mean ± S.E.M. and *P < 0.05, **P < 0.01 compared to control group.

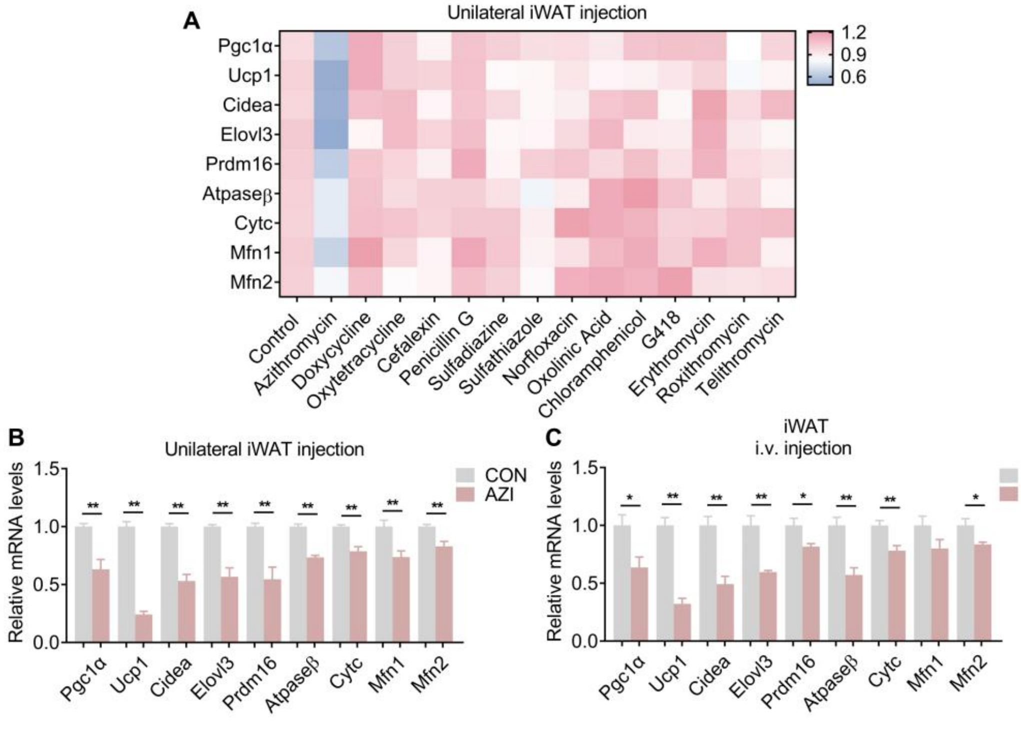

Figure S2. Azithromycin inhibited thermogenic and mitochondrial gene programs of iWAT in mice via iWAT or i.v. injection

(A) Heat map showing thermogenic and mitochondrial gene programs in iWAT of mice treated with different antibiotics at 1 mg/kg and controls (5% DMSO in saline) by unilateral iWAT injection acutely on Monday, Wednesday and Friday for a week (n = 4 per group)

(B, C) mRNA levels of thermogenic and mitochondrial gene programs in iWAT of mice treated with or without AZI at 1 mg/kg (B) by unilateral iWAT injection, and 50 mg/kg (C) by intravenous injection on Monday, Wednesday and Friday for a week (n

= 4).

Data are presented as mean ± S.E.M. and *P < 0.05, **P < 0.01 compared to control group.

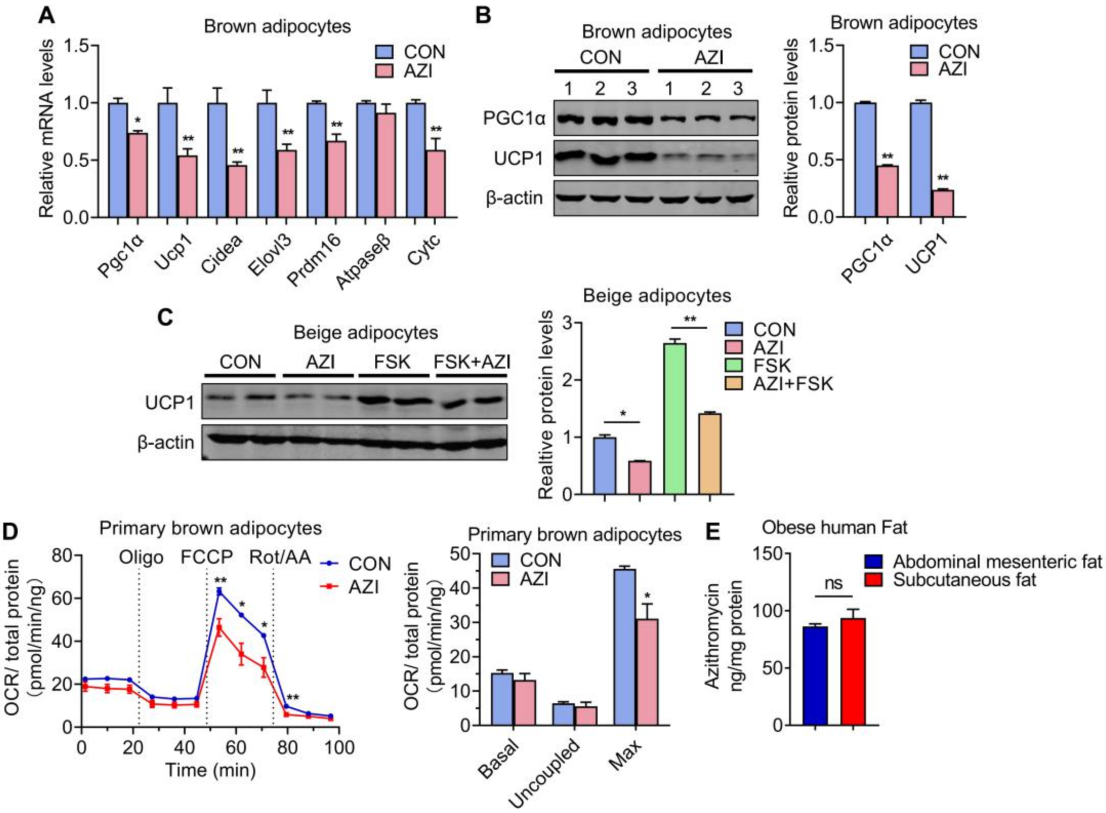

Figure S3. Azithromycin suppressed brown gene programs in immortal brown/beige adipocytes

(A, B) mRNA levels of thermogenic and mitochondrial gene programs (A) and UCP1 and PGC1α protein levels (B) in brown immortal adipocytes treated with or without AZI at 5 μM for 24 and 48 h (n = 3).

(C) UCP1 protein levels of immortal beige adipocytes treated with or without AZI at 5 μM for 48 h and forskolin (FSK, 10 μM) for 24 h (n = 3).

(D) Oxygen consumption rate (OCR) of primary brown adipocytes treated with or without AZI at 5 μM for 24 h. (n = 5 independent experiments)

(E) AZI accumulation (ng/mg protein) in abdominal mesenteric region and subcutaneous fat from same individual obese (BMI > 30) patients.

Data are presented as mean ± S.E.M. and *$P < 0.05$, **$P < 0.01$ compared to control group.

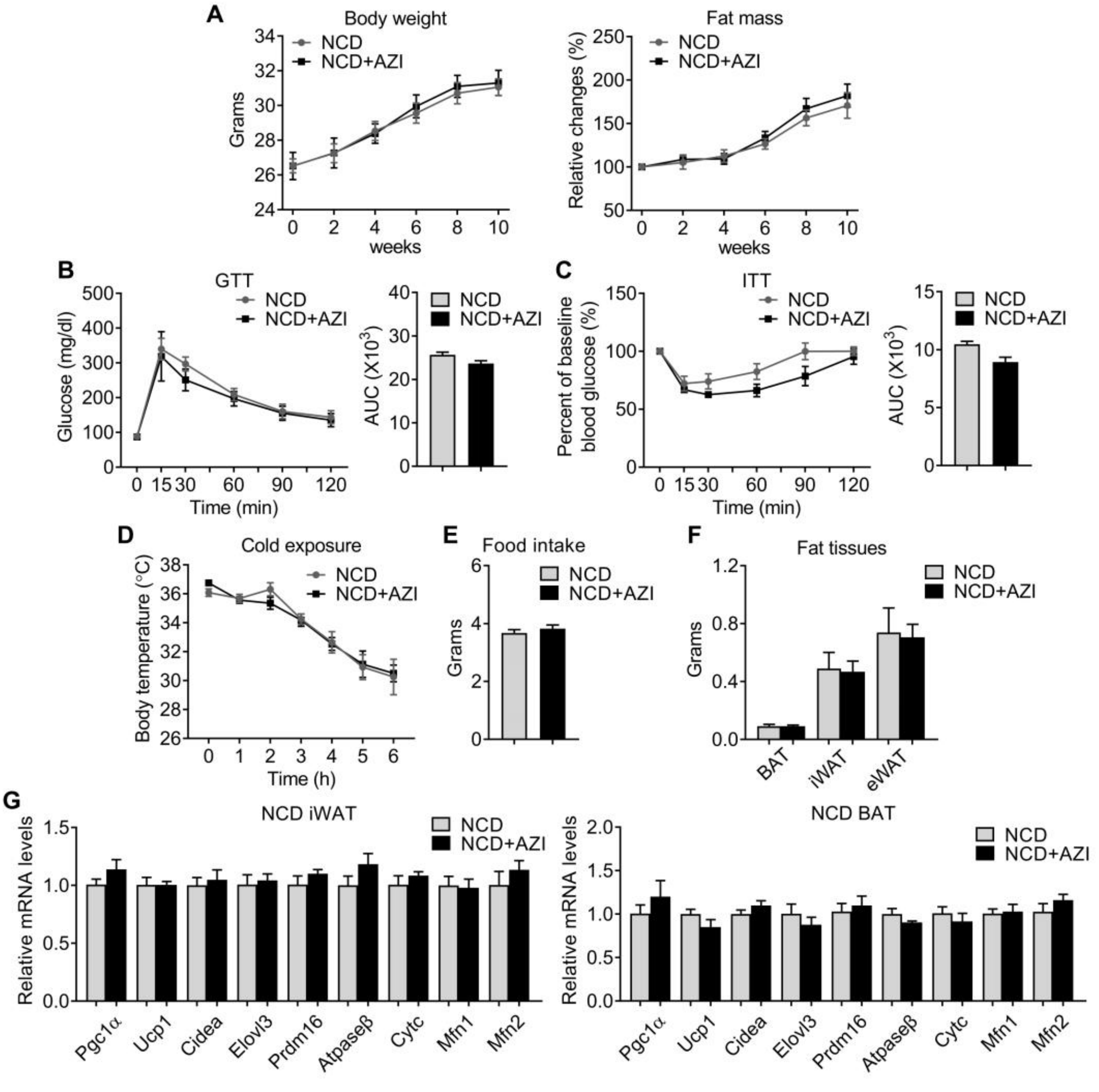

Figure S4. Azithromycin treatment did not alter metabolic performance in chow diet fed mice

(A-G) Metabolic performances of chow diet fed mice treated with or without AZI via drinking water (ABX is given throughout the whole process) (ABX: 1.0 g/L and AZI: 50 mg/kg/day) (n = 6).

(A) Body weight and relative change of fat mass; (B) Glucose tolerance (GTT) and area under the curve (AUC) analysis; (C) Insulin tolerance test (ITT) and AUC analysis; (D) Rectal temperatures of mice during 6 h cold exposure; (E) Food intake; (F) Tissue weights of brown (BAT), inguinal (iWAT) and epididymal (eWAT) fat pads; (G) mRNA levels of thermogenic and mitochondrial gene programs in BAT and iWAT

of mice.

Data are presented as mean ± S.E.M. and *P < 0.05, **P < 0.01 compared to control group.

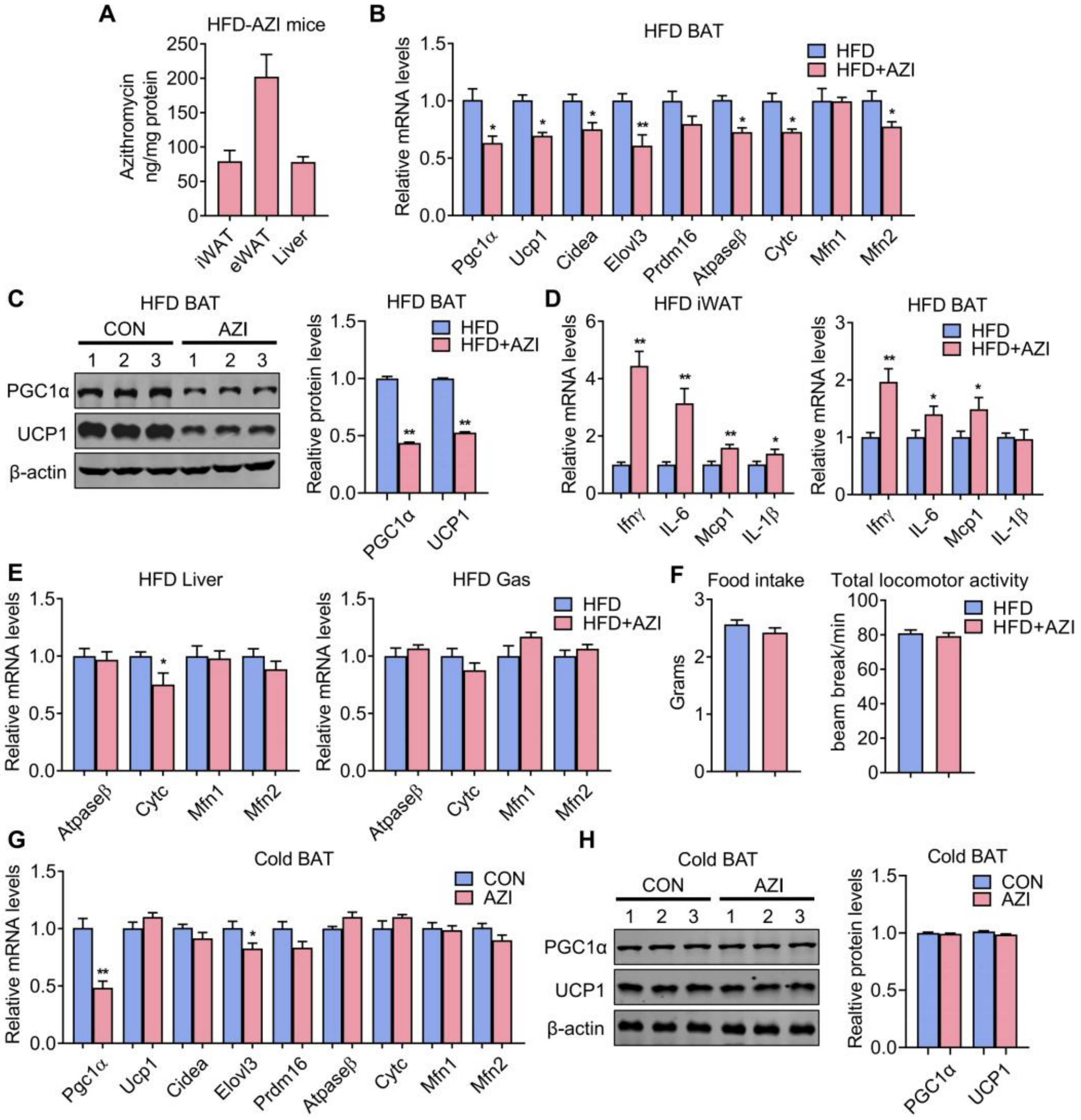

Figure S5. Gene programs in fat of HFD fed mice treated with or without Azithromycin

(A-H) Metabolic performances of HFD fed mice treated with or without AZI via drinking water for 12 weeks (ABX is given throughout the whole process, ABX: 1.0 g/L and AZI: 50 mg/kg/day, n = 6).

(A) AZI accumulation (ng/mg protein) in iWAT, eWAT and Liver of HFD mice under azithromycin treatment via drinking water.

(B, C) mRNA levels of thermogenic and mitochondrial gene programs (B) and UCP1 and PGC1α protein levels (C) in BAT.

(D) mRNA levels of inflammatory genes in BAT and iWAT.

(E) mRNA levels of mitochondrial gene programs in liver and gastrocnemius muscle.

(F) Food intake and total locomotor activity of mice.

(G, H) mRNA levels of thermogenic and mitochondrial gene programs (G) and UCP1 and PGC1α protein levels (H) in BAT of mice after 6 h cold exposure.

Data are presented as mean ± S.E.M. and $^{*}P < 0.05$, $^{**}P < 0.01$ compared to control group.

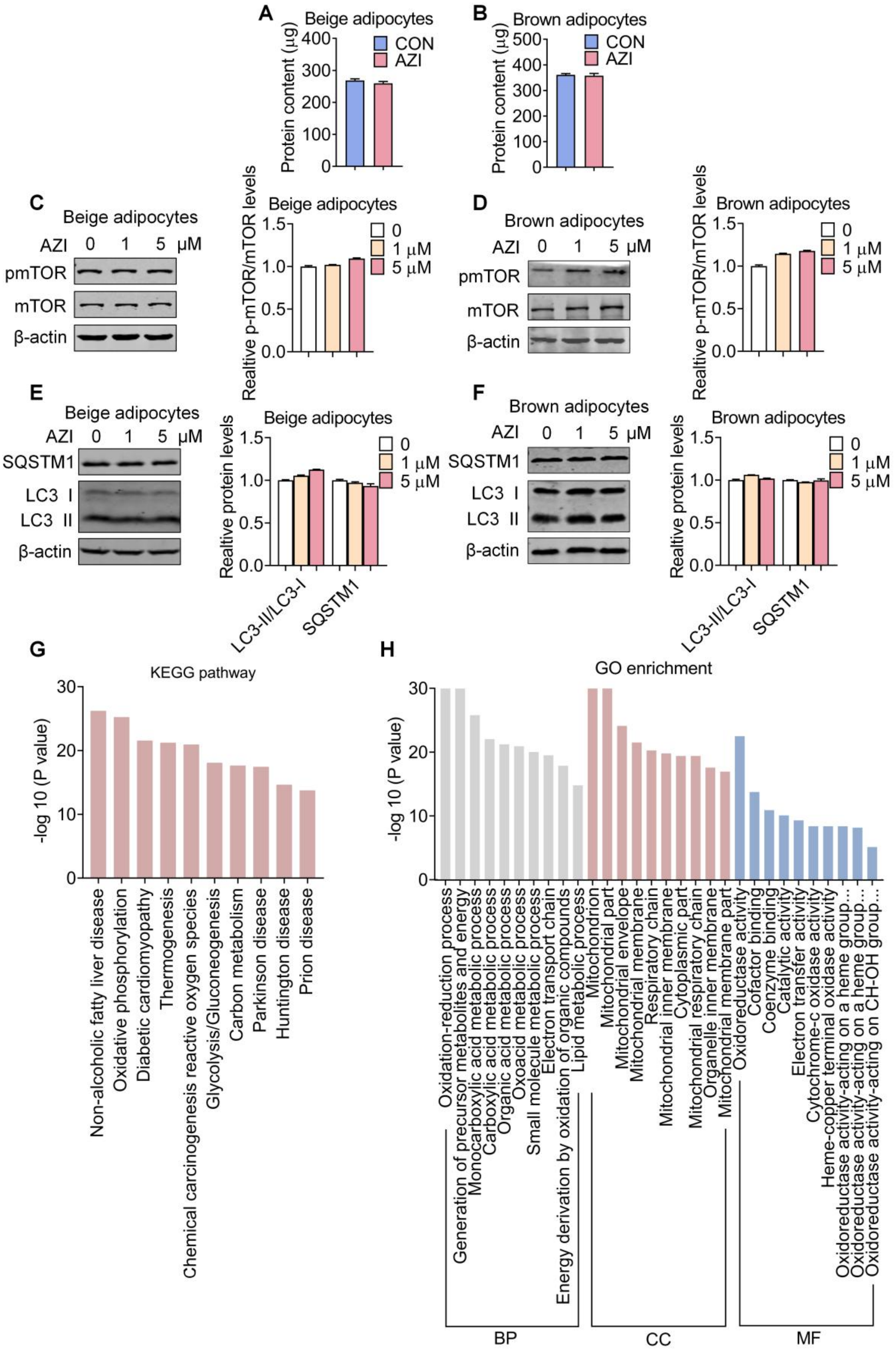

Figure S6. Azithromycin treatment damaged oxidative phosphorylation and mitochondria functionalities in immortal beige adipocytes

(A, B) Cellular protein content; (C, D) p-mTOR/mTOR protein levels; (E, F) SQSTM1 and LC3 II/I protein levels in immortal beige and brown adipocytes treated

with or without AZI at indicated concentration (0, 1 and 5 μM) for 48 h (n = 3).

(G) KEGG enrichment Barplot of metabolic pathway (Top10) from RNA-seq data with or without AZI (5 μM) treatment in beige adipocytes.

(H) GO enrichment Barplot of biological processes, cellular component and molecular function (Top10 of each classification) from RNA-seq data with or without AZI (5 μM) treatment in beige adipocytes.

Data are presented as mean ± S.E.M. and $*P < 0.05$, $**P < 0.01$ compared to control group.

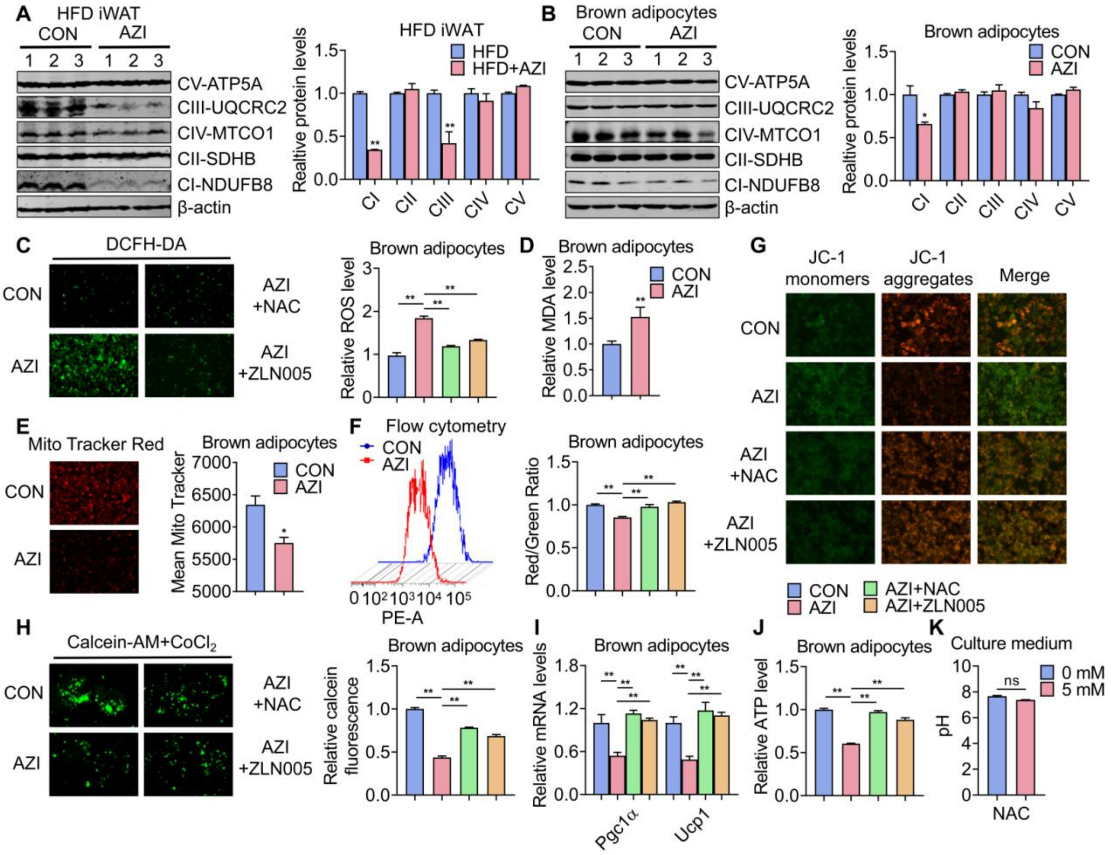

Figure S7. Azithromycin caused mitochondrial dysfunction and oxidative damage in immortal brown adipocytes

(A, B) Protein levels of major mitochondrial OXPHOS components in iWAT of AZI-treated mice under HFD (A) and immortal brown adipocytes (B) treated with or without AZI at 5 μM for 48 h (n = 3).

(C-J) Immortal brown adipocytes treated with control (CON), AZI (5 μM), AZI+NAC (5 mM) and AZI+ZLN005 (10 μM) (n = 3).

(C) Reactive oxygen species (ROS) staining and quantification; (D) Relative malondialdehyde (MDA) levels; (E, F) Mitotracker fluorescent staining and flow cytometry analysis of fluorescence intensity; (G) Mitochondrial membrane potential by JC-1 staining analysis; (H) Mitochondrial Permeability Transition Pore (mPTP) analysis; (I) Ucp1 and Pgc1α mRNA levels and (J) Relative ATP levels.

(K) pH levels after dissolving 5 mM NAC in culture medium (n = 3).

Data are presented as mean ± S.E.M. and *P < 0.05, **P < 0.01 compared to control group.

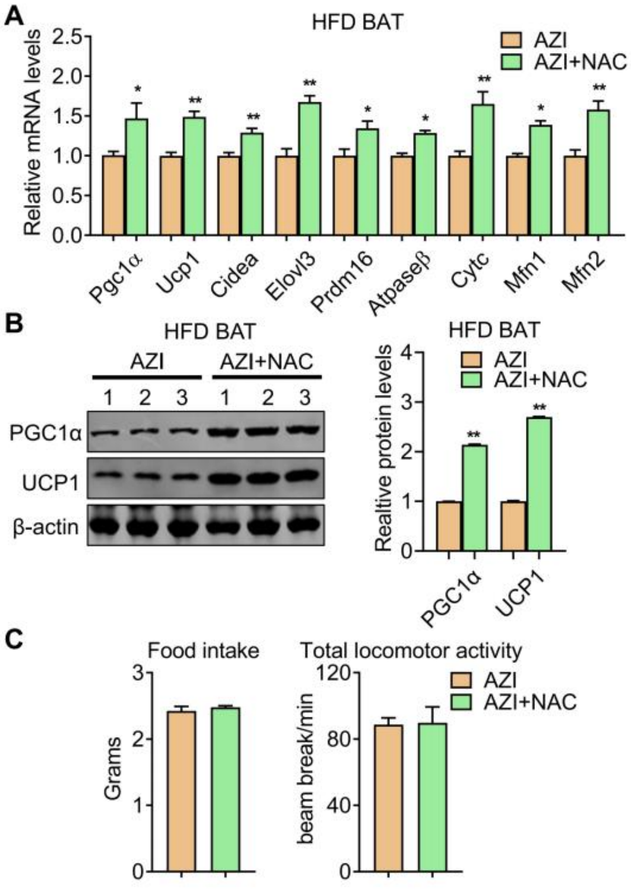

Figure S8. Gene programs in brown fat of HFD fed mice treated with Azithromycin or Azithromycin and N-Acetylcysteine

(A-C) Metabolic performances of HFD fed mice treated with AZI and AZI+NAC via drinking water for 12 weeks (ABX is given throughout the whole process, ABX: 1.0 g/L, AZI: 50 mg/kg/day and NAC:1.5 g/kg/day, n = 6).

(A, B) mRNA levels of thermogenic and mitochondrial gene programs (A) and UCP1

and PGC1α protein levels (B) in BAT.

(C) Food intake and total locomotor activity of mice.

Data are presented as mean ± S.E.M. and $*P < 0.05$, $**P < 0.01$ compared to control group.

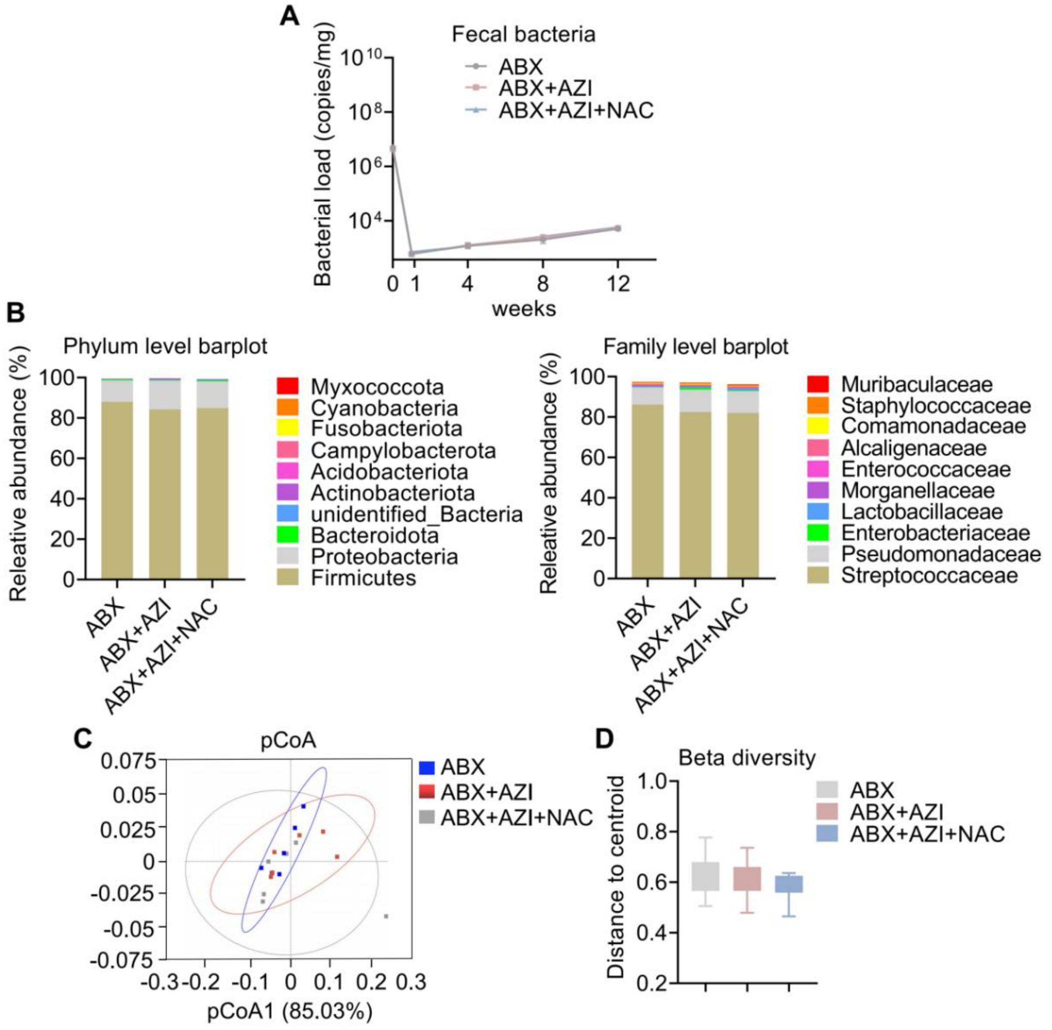

Figure S9. Microbiome composition in HFD fed mice treated with ABX, ABX+AZI and ABX+AZI+NAC via drinking water for 12 weeks

(A) Quantitative analysis of bacterial load in feces of HFD mice treated with ABX (1.0 g/L), ABX+AZI (50 mg/kg/day) and ABX+AZI+NAC (1.5 g/kg/day) via drinking water at 1, 4, 8 and 12 weeks (n = 6).

(B) Fecal microbiota composition at both phylum and family levels of all three groups (n = 6).

(C) PCoA plot based on weighted uniFrac distance of all three groups (wilcox rank-sum test, n = 6)

(D) Beta diversity based on unweighted uniFrac distance of all three groups (tukey rank-sum test, n = 6).

Data are presented as mean ± S.E.M. and $*P < 0.05$, $**P < 0.01$ compared to control group.

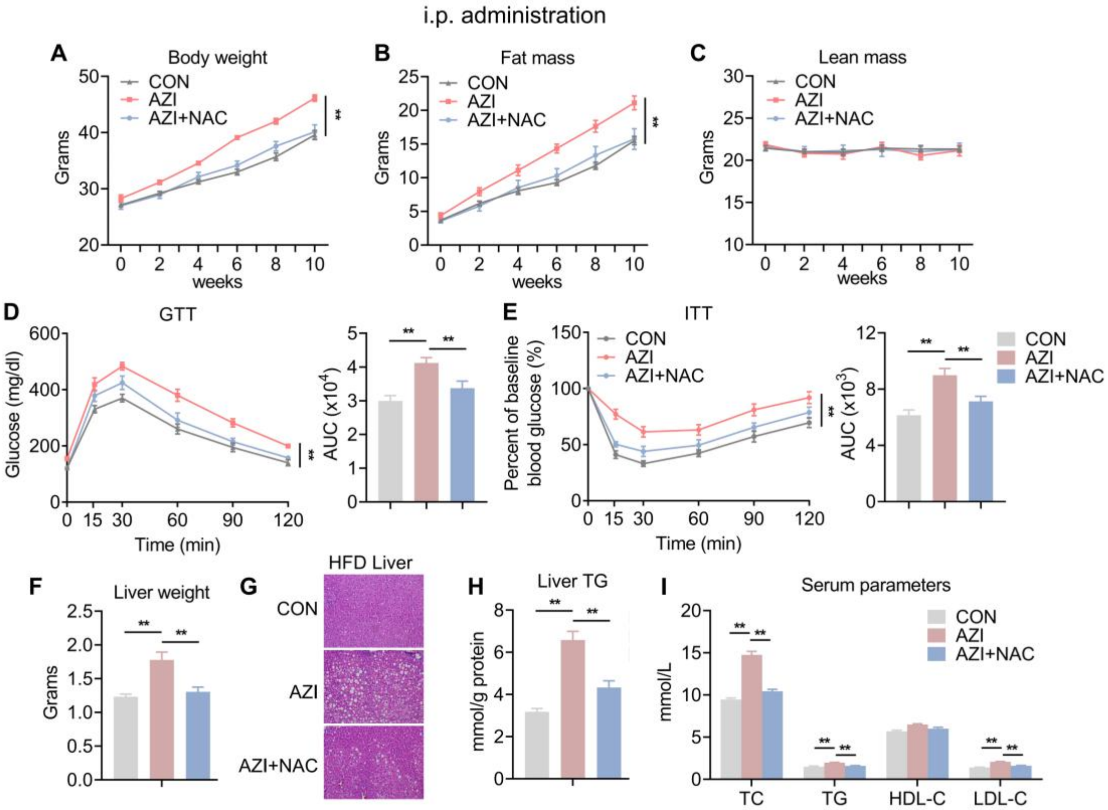

Figure S10. Intraperitoneal delivery of control, AZI and NAC in mice recapitulated metabolic performances of those via drinking water

(A-I) Metabolic performances of HFD fed mice i.p. injected with control (CON), AZI, AZI+NAC for 10 weeks. (AZI: 50 mg/kg, NAC: 100 mg/kg, n = 6).

(A) Body weight; (B) Fat mass; (C) Lean mass; (D) Glucose tolerance test (GTT) and area under the curve (AUC) analysis; (E) Insulin tolerance test (ITT) and AUC analysis; (F) Liver weight, (G) Representative H&E staining of liver; (H) Liver triglycerides levels and (I) Serum parameters.

Data are presented as mean ± S.E.M. and $*P < 0.05$, $**P < 0.01$ compared to control

group.

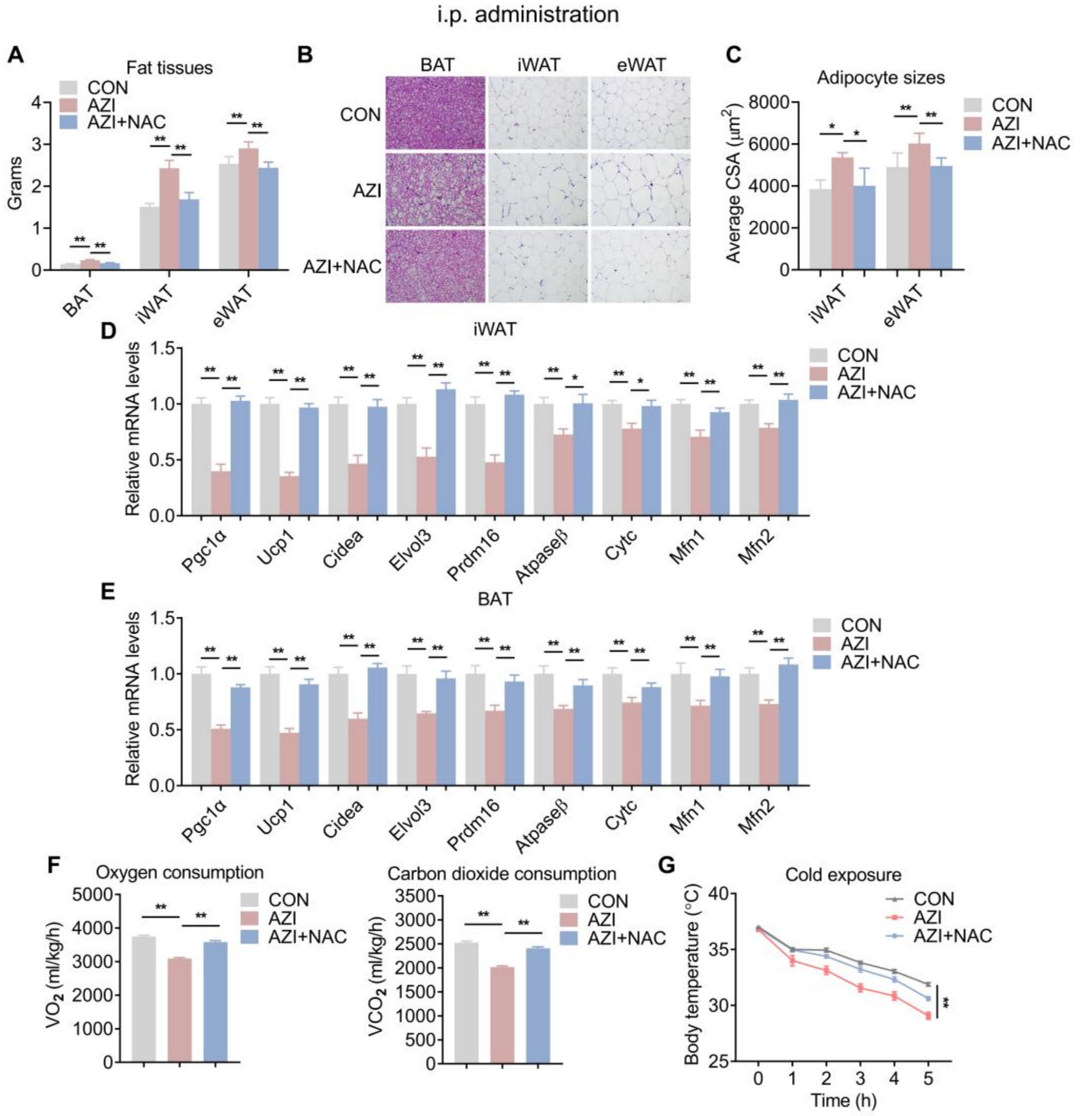

Figure S11. Intraperitoneal delivery of control, AZI and NAC in mice recapitulated fat biology and energy metabolism of those via drinking water

(A-I) Fat biology and energy metabolism of HFD fed mice i.p. injected with control (CON), AZI, AZI+NAC for 10 weeks. (AZI: 50 mg/kg, NAC: 100 mg/kg, n = 6).

(A) Tissue weights of brown (BAT), inguinal (iWAT) and epididymal (eWAT) fat pads.

(B, C) Representative images of H&E staining of fat tissues (B) and quantitative analysis of adipocyte sizes (C) of iWAT and eWAT.

(D, E) mRNA levels of thermogenic and mitochondrial gene programs in iWAT (D) and BAT (E).

(F) Energy expenditure was determined as oxygen consumption and carbon dioxide consumption.

(G) Rectal temperatures of mice during 5 h cold exposure.

Data are presented as mean ± S.E.M. and *$P < 0.05$, **$P < 0.01$ compared to control group.

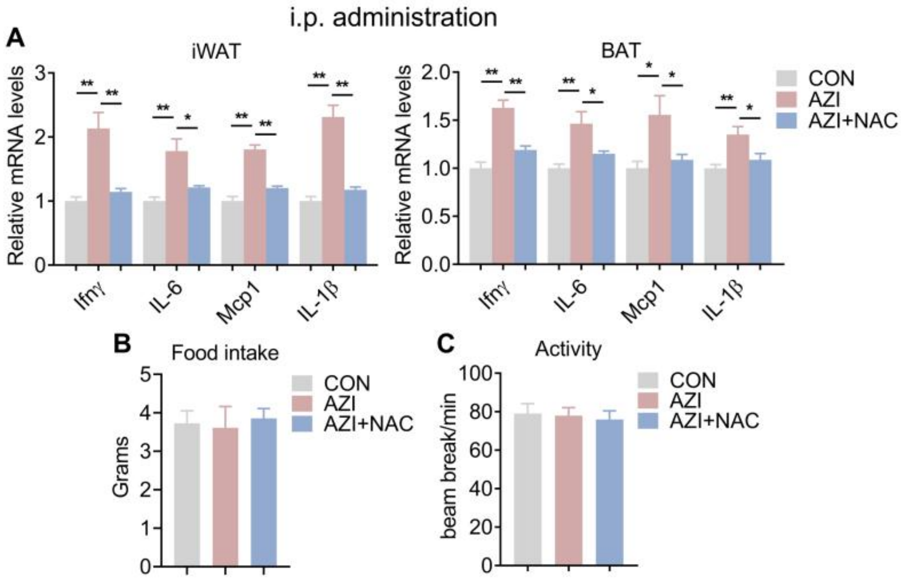

Figure S12. Inflammatory gene programs in iWAT or BAT of HFD fed mice treated with control, AZI and NAC

(A) mRNA levels of inflammatory genes in BAT and iWAT of HFD fed mice i.p. injected with control (CON), AZI, AZI+NAC for 10 weeks. (AZI: 50 mg/kg, NAC: 100 mg/kg, n = 6).

(B, C) Food intake and total locomotor activity of mice (n = 6).

Data are presented as mean ± S.E.M. and *$P < 0.05$, **$P < 0.01$ compared to control group.

Table S1. qPCR primers for gene expression level analysis in mouse and human

Gene name	Primer sequence (5′ → 3′)	
m36B4	Forward	AGATTCGGGATATGCTGTTGGC
	Reverse	TCGGGTCCTAGACCAGTGTTC
mUcp1	Forward	GGCCCTTGTAAACAACAAAATAC

	Reverse	GGCAACAAGAGCTGACAGTAAAT
mPgc1α	Forward	ACCATGACTACTGTCAGTCACTC
	Reverse	GTCACAGGAGGCATCTTTGAAG
mCidea	Forward	TGACATTCATGGGATTGCAGAC
	Reverse	CGAGCTGGATGTATGAGGGG
mElovl3	Forward	TTCTCACGCGGGTTAAAAATGG
	Reverse	TCTCGAAGTCATAGGGTTGCAT
mPrdm16	Forward	CCACCAGCGAGGACTTCAC
	Reverse	GGAGGACTCTCGTAGCTCGAA
mAtpaseβ	Forward	GACATGGGCACAATGCAGG
	Reverse	GCAGGGTCAGTCAGGTCATCA
mCytC	Forward	AAATCTCCACGGTCTGTTCGG
	Reverse	GGGTATCCTCTCCCCAGGTG
mMfn1	Forward	AACCGAGAAGCTGCAGATGA
	Reverse	TCAACTTGTTGGCACAGTCG
mMfn2	Forward	AGGCCTTCCTCCTCACAGAG
	Reverse	AGTTGGGCCACATCACACTC
mIl-1β	Forward	AAATACCTGTGGCCTTGGGC
	Reverse	CTTGGGATCCACACTCTCCAG
mIl-6	Forward	TAGTCCTTCCTACCCCAATTTCC
	Reverse	TTGGTCCTTAGCCACTCCTTC
mMcp1	Forward	TTAAAAACCTGGATCGGAACCAA
	Reverse	GCATTAGCTTCAGATTTACGGGT
mIfnγ	Forward	ACAGCAAGGCGAAAAAGGATG
	Reverse	TGGTGGACCACTCGGATGA
hGapdh	Forward	GAAGGTGAAGGTCGGAGT
	Reverse	GAAGATGGTGATGGGATTTC
hUcp1	Forward	GGCTACTTGGGCTATTGTAAAGG
	Reverse	CAGTTTCTCCGACAACTTTCTCT
hPgc1α	Forward	TCTGAGTCTGTATGGAGTGACAT
	Reverse	CCAAGTCGTTCACATCTAGTTCA

Table S2. Alpha diversity and Beta diversity of mice fecal microbiota

P value	Alpha diversity		Beta diversity
Group pair	Observed species	Shannon index	
ABX — ABX+AZI	0.9583	0.3534	0.6766
ABX+AZI — ABX+AZI+NAC	0.3284	0.8753	0.5095
ABX — ABX+AZI+NAC	0.3044	0.2814	0.1341
